# SARS‐CoV‐2 Spike Protein as a Target of the COVID‐19 Vaccine Disrupts Insulin Signaling in Type 2 Diabetes

**DOI:** 10.1002/mco2.70469

**Published:** 2025-11-02

**Authors:** Lixiang Zhai, Min Zhuang, Hoi Ki Wong, Chengyuan Lin, Haoran Ying, Jialing Zhang, Gengyu Bao, Yijing Zhang, Shujun Xu, Jingyuan Luo, Shuofeng Yuan, Hoi Leong Xavier Wong, Zhao‐Xiang Bian

**Affiliations:** ^1^ Centre For Chinese Herbal Medicine Drug Development Hong Kong Baptist University Hong Kong SAR China; ^2^ School of Chinese Medicine Hong Kong Baptist University Hong Kong SAR China; ^3^ Department of Microbiology Li Ka Shing Faculty of Medicine The University of Hong Kong Hong Kong SAR China

**Keywords:** COVID‐19, diabetes, insulin sensitivity, SARS‐CoV‐2, vaccine

## Abstract

Diabetes is associated with an increased risk of coronavirus disease 2019 (COVID‐19)‐associated morbidity and mortality. COVID‐19 vaccines substantially reduce the risk of serious COVID‐19 outcomes, making them important for individuals with diabetes. However, the effects of the COVID‐19 vaccines on glucose control in patients with diabetes remain unclear. Here, we demonstrate that COVID‐19 vaccine boosters impair insulin sensitivity in both mice and patients with type 2 diabetes (T2D). In mice, the administration of four vaccine doses elevated the levels of SARS‐CoV‐2 spike protein and impaired glucose tolerance and insulin sensitivity. Mechanistically, we showed that the SARS‐CoV‐2 spike protein, guided by the mRNA COVID‐19 vaccine, interferes with insulin signaling by binding to angiotensin‐converting enzyme 2, TLR4, and ER. We found that 66% of T2D patients exhibited aggravated insulin resistance to booster shots of the mRNA COVID‐19 vaccine. Furthermore, treatment with metformin improved insulin signaling variations induced by COVID‐19 vaccine boosters in mice. These findings indicate that COVID‐19 vaccine boosters impair insulin sensitivity in T2D and that metformin may mitigate these effects. These results maintain the risk–benefit ratio in favor of COVID‐19 vaccination for the prevention of severe clinical outcomes, yet highlight the need for close glycemic monitoring of patients with diabetes after receiving mRNA COVID‐19.

## Graphical abstract

SARS Cov‐2 spike protein disrupts insulin signaling in type 2 diabetes as a target of the COVID‐19 vaccine in subject with type 2 diabetes (T2D), while metformin improved the insulin signaling variations induced by mRNA COVID‐19 vaccine boosters in mice.

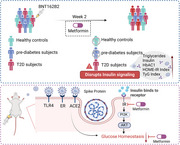

## 1 Introduction

Diabetes mellitus (DM), commonly referred to as diabetes, is a chronic metabolic disorder characterized by high blood glucose levels due to insufficient insulin production (type 1 diabetes) or insulin resistance (type 2 diabetes [T2D]). Diabetes is one of the most prevalent metabolic diseases worldwide, affecting approximately 10% of the global population [1]. The impact of diabetes extends beyond the direct implications of the disease itself, as it predisposes individuals to a myriad of complications [2]. Diabetes can lead to various health complications. For instance, cardiovascular diseases are notably more common among patients with diabetes, and these conditions contribute to the development of atherosclerosis, increasing the risk of heart attack and stroke. Diabetic neuropathy is another frequent complication in which high blood sugar levels cause damage to nerve fibers, particularly in the legs and feet, leading to numbness, tingling, or pain. Nephropathy, which is kidney damage resulting from diabetes, can progress to kidney failure, a life‐threatening condition.

The coronavirus disease 2019 (COVID‐19) pandemic, caused by severe acute respiratory syndrome coronavirus 2 (SARS‐CoV‐2), has brought challenges for people with diabetes, as they have an increased risk of more severe symptoms and complications from COVID‐19 [3]. Patients with diabetes are more susceptible to an array of complications from SARS‐CoV‐2, including a higher likelihood of requiring intensive care and mechanical ventilation and a greater risk of death than the general population [4, 5]. Diabetes is associated with an impaired immune response; therefore, COVID‐19 infections can lead to more severe and prolonged illness in patients with diabetes. Recent data indicate that people with diabetes and metabolic dysfunction not only experience more severe outcomes from COVID‐19 infection, but that COVID‐19 can also trigger acute metabolic complications, including diabetic ketoacidosis and hyperglycemia [6]. In individuals with COVID‐19 infection, hyperglycemia is primarily caused by insulin resistance, which is associated with poor outcomes and persists even after clinical recovery [7]. Although the exact mechanisms underlying these connections are not fully understood, they are likely to involve the angiotensin‐converting enzyme 2 (ACE2) receptor. A deeper understanding of the bidirectional interactions between diabetes and COVID‐19 is essential for effectively managing individuals with diabetes or those at a high risk of metabolic dysfunction [8].

COVID‐19 vaccination has been shown to reduce the risk of symptomatic infections, hospitalization rates, and mortality from COVID‐19 caused by the SARS‐CoV‐2 virus [9]. Moreover, large‐scale vaccination data have demonstrated a substantial decrease in hospitalization and mortality rates due to COVID‐19 infection in the diabetic population [10]. Collectively, these data strongly suggest that the benefits of COVID‐19 vaccination significantly outweigh the risks, particularly for individuals with preexisting health conditions such as diabetes. Thus, COVID‐19 vaccination has become a critical defense mechanism in individuals with diabetes. By bolstering the immune response to SARS‐CoV‐2, COVID‐19 vaccines help prevent hyper‐inflammatory reactions and severe respiratory complications that can arise from COVID‐19 infection.

Recognizing the advantages of the COVID‐19 vaccine, it is imperative to elucidate any unforeseen effects, especially in individuals with pre‐existing metabolic conditions. Moreover, many countries use booster doses, including three and four doses of the COVID‐19 vaccine, to maintain immune protection [11]; thus, there is a need for ongoing surveillance to track the adverse outcomes of COVID‐19 vaccination in individuals with diabetes. As the interplay between COVID‐19 vaccination and diabetes is not entirely understood, it is critical to conduct both basic science and clinical research to understand the impact of COVID‐19 vaccination on glucose control in patients with diabetes.

## 2 Results

### 2.1 mRNA COVID‐19 Vaccine Impairs Insulin Signaling in Healthy Mice

**FIGURE 1:**
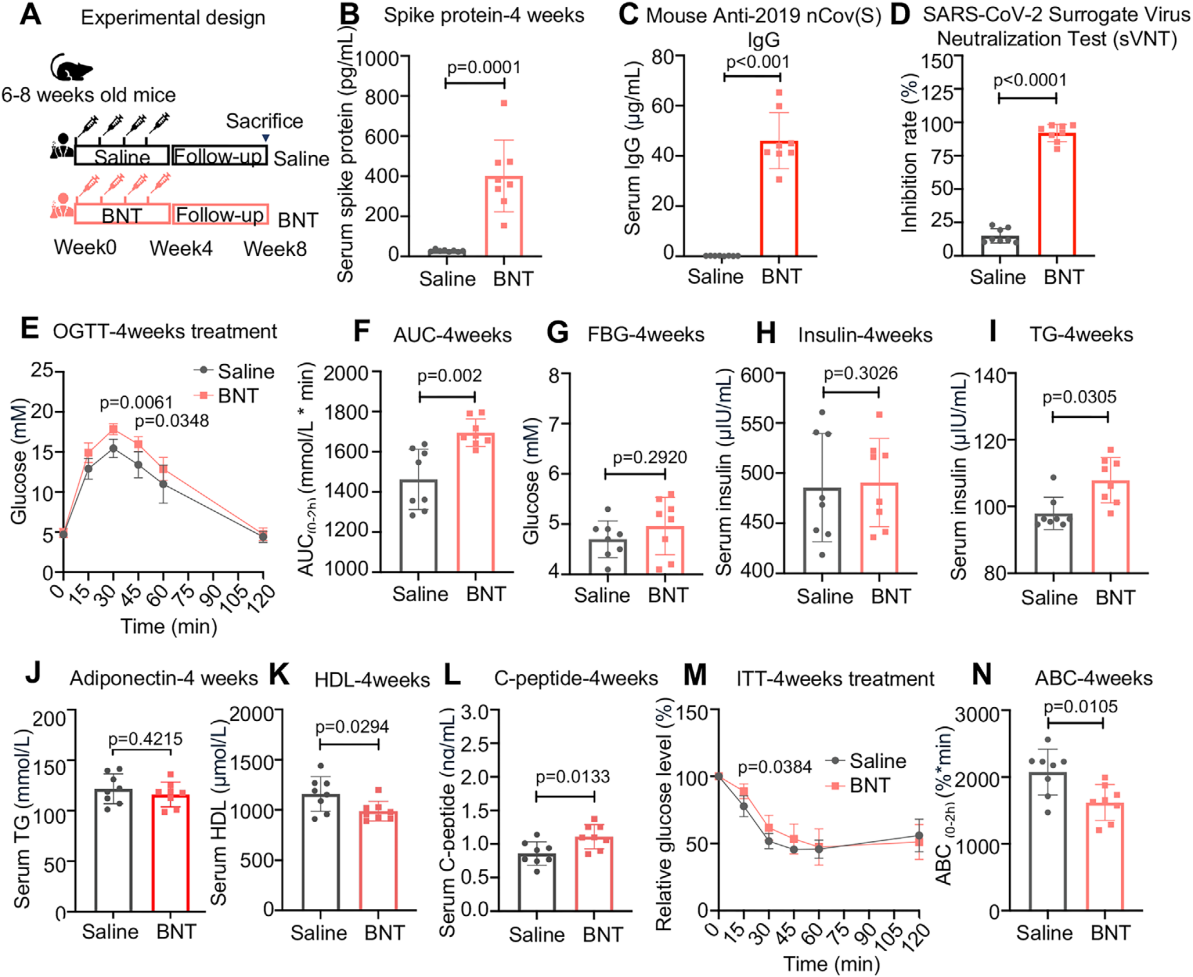
mRNA COVID‐19 vaccine impairs glucose intolerance in mice. (A) Experimental design for mRNA COVID‐19 vaccination in wild‐type mice. (B–D) SARS‐CoV‐2 spike protein, SARS‐CoV‐2 spike (trimer) IgG, and SARS‐CoV‐2 sVNT in serum samples from mice following treatment of BNT mRNA COVID‐19 vaccine (4.5 µg/kg) for 4 weeks (*n* = 8/group; statistical significance determined by two‐tailed *t*‐tests). (E–F) OGTT and area under curve (AUC) indices in mice following treatment of BNT mRNA COVID‐19 vaccine (4.5 µg/kg) for 4 weeks (*n* = 8/group; OGTT determined by two‐way ANOVA, AUC determined by two‐tailed *t*‐tests). (G–L) Fasting blood glucose (FBG), fasting insulin, fasting triglyceride (TG), adiponectin, high‐density lipoprotein cholesterol (HDL‐C), and C‐peptide levels in serum samples from mice following treatment of BNT mRNA COVID‐19 vaccine (4.5 µg/kg) for 4 weeks (*n* = 8/group; statistical significance determined by two‐tailed *t*‐tests). (M and N) ITT and area below curve (ABC) indices in mice following treatment of BNT mRNA COVID‐19 vaccine (4.5 µg/kg) for 4 weeks (*n* = 8/group; ITT determined by two‐way ANOVA, ABC determined by two‐tailed *t*‐tests).

**FIGURE 2:**
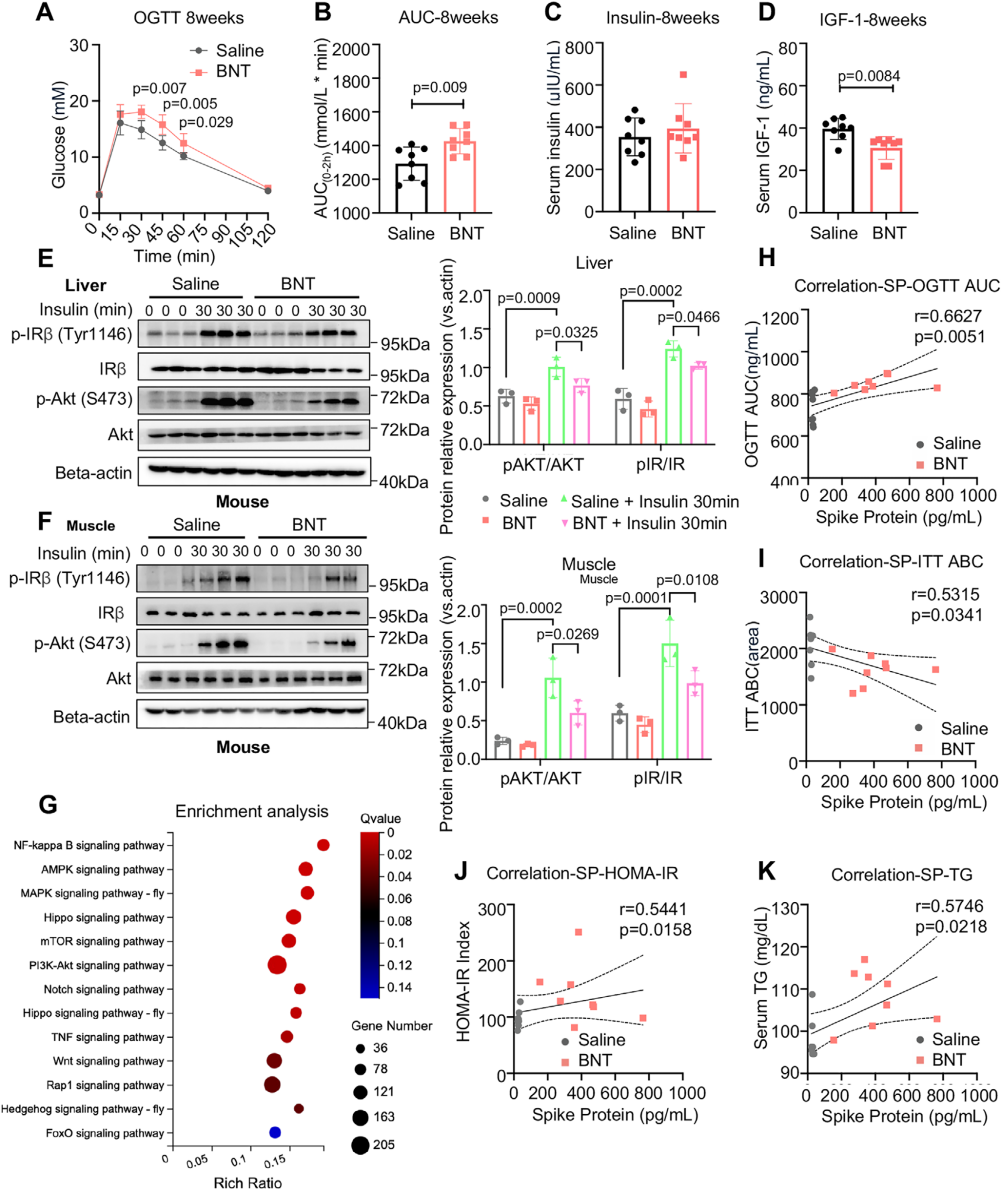
mRNA COVID‐19 vaccine disrupts insulin signaling in mice. (A and B) OGTT and area under curve (AUC) indices in mice following treatment of BNT mRNA COVID‐19 vaccine (4.5 µg/kg) for 8 weeks (*n* = 8/group) (OGTT determined by two‐way ANOVA, AUC determined by two‐tailed t‐tests). (C and D) Insulin and IGF‐1 in serum samples from mice following treatment of BNT mRNA COVID‐19 vaccine (4.5 µg/kg) for 8 weeks (*n* = 8/group; statistical significance determined by two‐tailed *t*‐tests). (E and F) Western blot (and quantification) demonstrated the effects of BNT mRNA COVID‐19 vaccine (4.5 µg/kg) on insulin receptor (IR) and AKT phosphorylation in the liver and muscle lysates from mice (*n* = 3/group; determined by two‐tailed one‐way ANOVA test). (G) KEGG‐based enrichment analysis of signaling pathways in liver lysates of mice following treatment with the BNT mRNA COVID‐19 vaccine (*n* = 3/group; statistical significance determined by two‐tailed *t*‐test). (H–K) Spearman's correlation analysis between serum spike protein level and the OGTT‐AUC, ITT‐ABC, and HOMA‐IR indices in mice following treatment of BNT mRNA COVID‐19 vaccine (4.5 µg/kg) or saline (*n* = 8/group; statistical significance determined by two‐tailed tests).

To study the effect of COVID‐19 vaccination on glucose control, we first designed a study to evaluate the changes in glucose control and insulin sensitivity in healthy mice that received the mRNA COVID‐19 vaccine (BNT162b2) weekly (Figure 1A). Compared to mice treated with saline, mice treated with the four doses of the mRNA COVID‐19 vaccine (week 4) exhibited immune responses characterized by the elevation of the SARS‐CoV‐2 spike protein and IgG antibodies against the SARS‐CoV‐2 trimeric spike protein, and the neutralizing abilities of SARS‐CoV‐2 protein significantly increased in the serum (*p* = 0.001, Figure 1B–D). Interestingly, we found that mice administered four doses of the COVID‐19 vaccine (week 4) exhibited impaired glucose tolerance, as evaluated by the oral glucose tolerance test (OGTT), fasting blood glucose (FBG), and serum insulin levels (*p* < 0.05, Figure 1E,F; n.s. for Figure 1G,H). Serum triglyceride (TG) and C‐peptide levels were significantly elevated, and high‐density lipoprotein (HDL) levels were significantly decreased, in mice treated with the COVID‐19 vaccine (week 4) (*p* < 0.05, in all cases, Figure 1J; n.s., Figure 1I–L), indicating an increased risk of metabolic disorders. Coupled with impaired glucose tolerance, the reduction in blood glucose in response to the insulin challenge in the insulin tolerance test (ITT) was also significantly reduced in mice with COVID‐19 vaccination (week 4) (*p* < 0.05, in all cases, Figure 1M,N). In contrast, there were no significant changes in the OGTT results during the first 3 weeks (Figure S1A–D). Notably, we showed that glucose intolerance in COVID‐19 vaccine‐treated mice persisted after 4 weeks without vaccination (week 8) (*p* < 0.05, Figure 2A–D). In line with the ITT results, the level of insulin‐induced Akt phosphorylation in insulin‐sensitive tissues was significantly reduced in mice at week 8 after COVID‐19 vaccination (*p* < 0.05, Figure 2E,F). Subsequently, we analyzed the transcriptome of liver tissues in mice vaccinated with COVID‐19 vaccines at week 8. The enrichment analysis revealed that COVID‐19 vaccine boosters led to a dramatic activation of nuclear factor kappa‐light‐chain‐enhancer of activated B cells (NF‐κB), AMP‐activated protein kinase (AMPK), and mitogen‐activated protein kinase (MAPK) signaling pathways (Figure 2G and Table S1). In addition, serum SARS‐CoV‐2 spike protein levels were positively correlated with the glucose intolerance index, insulin resistance index, and serum TG levels in mice vaccinated with COVID‐19 (Figure 2H–K), suggesting that the SARS‐CoV‐2 spike protein, as stimulated by COVID‐19 vaccination, is linked with the aggravation of insulin resistance. Taken together, these results suggest that the glucose intolerance induced by the COVID‐19 vaccine is mediated by impaired insulin sensitivity rather than impaired insulin secretion in mice. To compare the results of the mRNA COVID‐19 vaccine, we administered an inactivated COVID‐19 vaccine (CoronaVac, Sinovac) to healthy mice. Mice that received four doses of this vaccine exhibited no significant impairments in glucose tolerance, as assessed by OGTT (Figure S2A–D).

### 2.2 SARS‐CoV‐2 Spike Protein Impairs Insulin Signaling via Multiple Signaling Pathways

**FIGURE 3:**
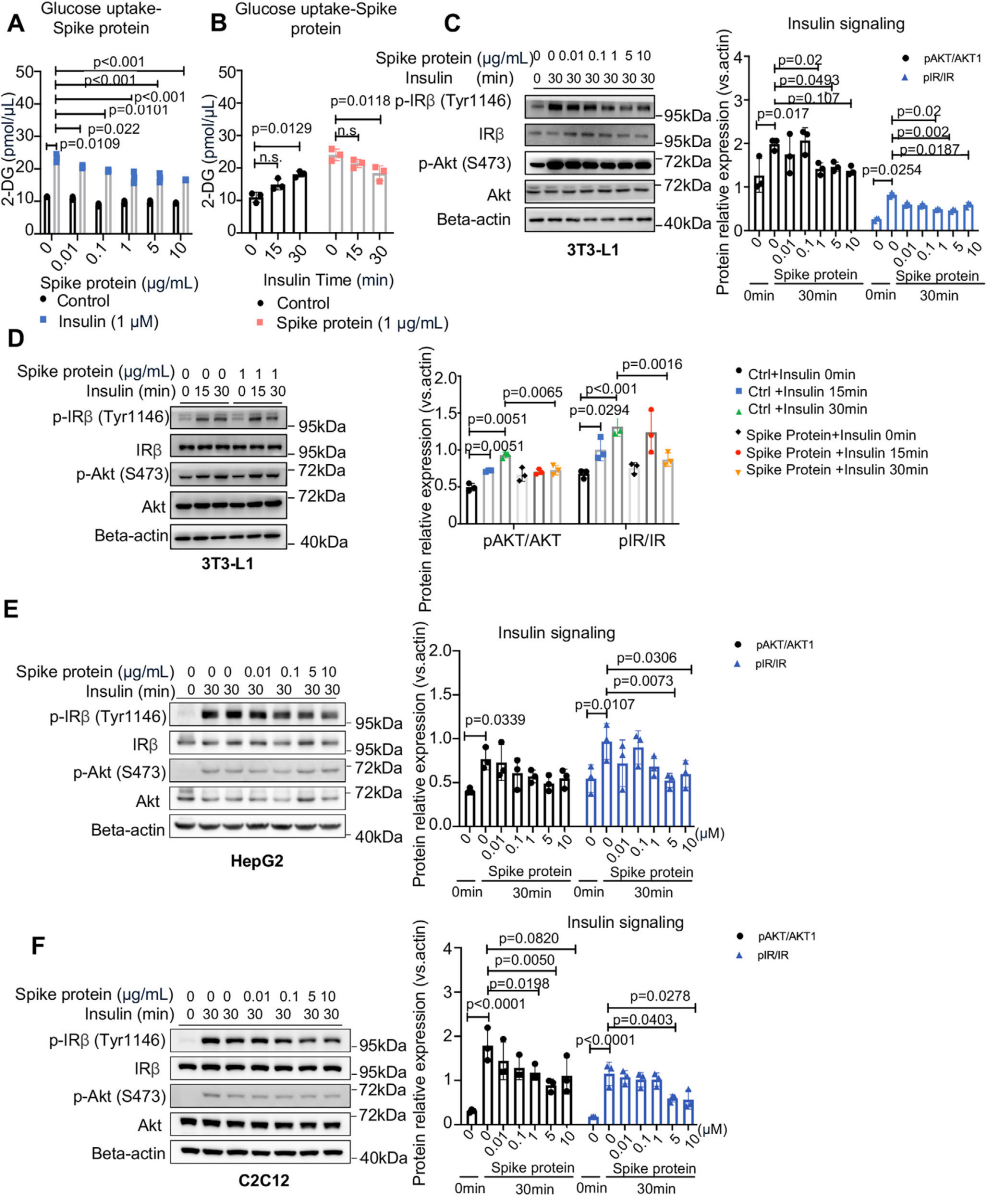
SARS‐CoV‐2 spike protein impairs insulin signaling in 3T3‐L1 cells. (A and B) Glucose uptake in 3T3‐L1 cells following treatment with SARS‐CoV‐2 spike protein (0.01–10 µg/mL) and insulin (1 µM) at the indicated dose and time (*n* = 3/group; determined by two‐tailed one‐way ANOVA test). (C and D) Western blot (and quantification) demonstrated the effect of spike protein (0.01–10 µg/mL) treatment on insulin receptor (IR) and AKT phosphorylation levels in 3T3‐L1 cells stimulated by insulin (100 nM) at the indicated dose and time (*n* = 3/group; statistical significance determined by two‐tailed one‐way ANOVA test). (E and F) Western blot (and quantification) demonstrated the effect of spike protein (0.01–10 µg/mL) on insulin receptor (IR) and AKT phosphorylation levels in HepG2 and C2C12 cells stimulated by insulin (100 nM) at the indicated dose and time (*n* = 3/group; statistical significance determined by two‐tailed one‐way ANOVA test).

The COVID‐19 vaccine can direct host cells to produce SARS‐CoV‐2 spike proteins, which internalize the ACE2 receptor for viral particle entry into the cells [12]. Moreover, the SARS‐CoV‐2 spike protein has been shown to bind to the Toll‐like receptor 4 (TLR4) and estrogen receptor (ER) in host cells [12, 13, 14]. These biological processes can affect multiple signaling systems, including the NF‐κB, AMPK, and MAPK signaling pathways, which may aggravate insulin resistance. We used 3T3‐L1 cells, an in vitro adipocyte cell model for studying insulin signaling, to assess the direct impact of SARS‐CoV‐2 spike proteins on insulin signaling by treating cells with SARS‐CoV‐2 spike protein in a physiological range. Notably, the insulin‐induced glucose uptake and phosphorylation of protein kinase B (Akt) and insulin receptor β‐subunit (IRβ) in 3T3‐L1 cells were significantly suppressed by the treatment of SARS‐CoV‐2 spike protein in a dose‐ and time‐dependent manner (all *p* < 0.05, Figure 3A–D). Similarly, treatment with the SARS‐CoV‐2 spike protein impaired insulin signaling in HepG2 and C2C12 cell lines in a similar manner to the 3T3‐L1 cells (*p* < 0.05, Figure 3E–F), revealing a direct inhibitory effect of the SARS‐CoV‐2 spike protein on insulin signaling in vitro.

**FIGURE 4:**
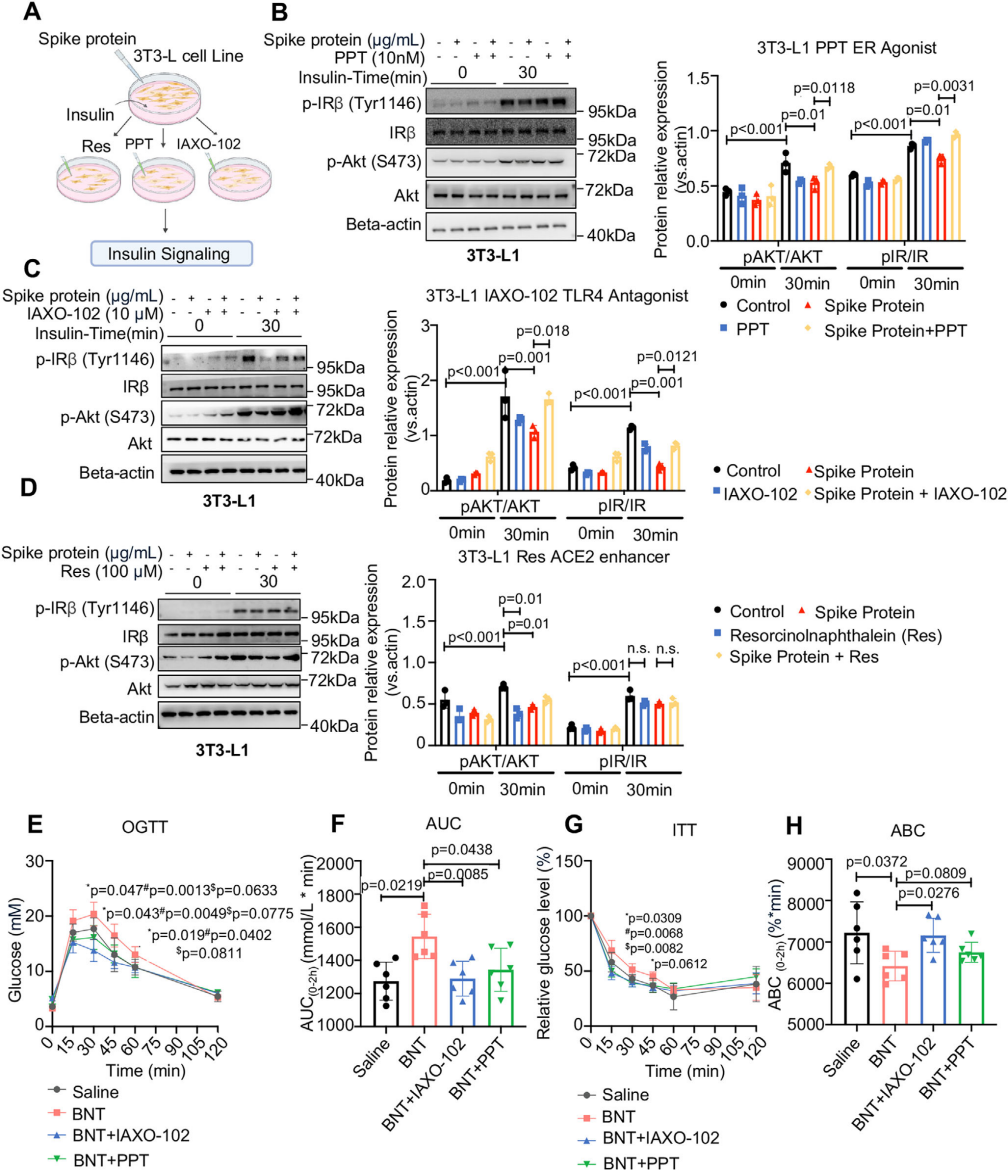
SARS‐CoV‐2 spike protein disrupts insulin sensitivity by binding to ACE2, TLR4, and ER. (A) Experimental design for treatment of SARS‐CoV‐2 spike protein in combination with a TLR4 antagonist, ER antagonist, and ACE2 enhancer on insulin signaling in 3T3‐L1 cells. (B) Western blot (and quantification) demonstrated the effect of SARS‐CoV‐2 spike protein (1 µg/mL) and the ER agonist PPT (10 nM) on IR and AKT phosphorylation in 3T3‐L1 cells (*n* = 3/group; statistical significance determined by two‐tailed one‐way ANOVA test). (C) Western blot (and quantification) demonstrated the effect of SARS‐CoV‐2 spike protein (1 µg/mL) and the TLR4 antagonist, IAXO‐102 (10 µM), on IR and AKT phosphorylation in 3T3‐L1 cells stimulated by insulin (1 µM) (*n* = 3/group; statistical significance determined by two‐tailed one‐way ANOVA test). (D) Western blot (and quantification) demonstrated the effect of SARS‐CoV‐2 spike protein (1 µg/mL) and the ACE2 enhancer resorcinolnaphthalein (100 µM) on IR and AKT phosphorylation levels in 3T3‐L1 cells stimulated by insulin (1 µM) (*n* = 3/group; statistical significance determined by two‐tailed one‐way ANOVA test). (E and F) OGTT and AUC indices in mice following treatment with the BNT mRNA COVID‐19 vaccine (4.5 µg/kg), ER agonist PPT (0.5 mg/kg), and TLR4 antagonist IAXO‐102 (3 mg/kg) (*n* = 6/group; OGTT determined by two‐way ANOVA, AUC determined by two‐tailed *t*‐tests). *Comparisons between the BNT and saline group, ^#^comparisons between the BNT and TLR4 antagonist group; ^$^comparisons between the BNT and ER antagonist group. (G and H) ITT and ABC indices in mice following treatment of BNT mRNA COVID‐19 vaccine, ER agonist PPT (0.5 mg/kg), and TLR4 antagonist IAXO‐102 (3 mg/kg) (*n* = 6/group; ITT determined by two‐way ANOVA, ABC determined by two‐tailed t‐tests). *Comparisons between BNT group and saline group. ^#^Comparisons between the BNT group and TLR4 antagonist group. ^$^Comparisons between the BNT group and ER antagonist group.

Accordingly, we investigated whether the insulin‐desensitizing effect of SARS‐CoV‐2 spike proteins is mediated by their binding to the host cell receptors ACE2, TLR4, and ER. We first noted that the mRNA expression of *Ace2*, *Ers1*, and *Ers2* was significantly changed after treatment with the SARS‐CoV‐2 spike protein, but *Tlr4* expression was not affected by the SARS‐CoV‐2 spike protein in 3T3‐L1 cells (*p* < 0.05, Figure S3A–D). We then determined the regulatory role of ACE2, TLR4, and ER in insulin signaling alterations induced by the SARS‐CoV‐2 spike protein using the TLR4 antagonist IAXO‐102, ER agonist PPT, and ACE2 enhancer resorcinol naphthalein (Figure 4A). Interestingly, we found that blockade of TLR4 using IAXO‐102 and activation of the ER using PPT largely abrogated the inhibitory effects of the SARS‐CoV‐2 spike protein on insulin signaling in 3T3‐L1 cells (*p* < 0.05, Figure 4B,C). We also showed that the activation of ACE2 using either the SARS‐CoV‐2 spike protein or the ACE2 enhancer resorcinol naphthalein altered insulin signaling affected by the SARS‐CoV‐2 spike protein (*p* < 0.05, Figure 4D). These results suggest that the SARS‐CoV‐2 spike protein binds to ACE2, TLR4, and ER to impair insulin signaling through TLR4 and ER.

To investigate the physiological relevance of our findings obtained from in vitro studies, we treated wild‐type mice with the mRNA COVID‐19 vaccine to induce a surge in circulating SARS‐CoV‐2 spike protein with an ER agonist (PPT) and a TLR4 antagonist (IAXO‐102). In line with in vitro results, ER activation or TLR4 inhibition effectively alleviated the impairment of insulin sensitivity and glucose tolerance induced by the COVID‐19 vaccine in mice (*p* < 0.05, Figure 4E–H). These results demonstrate the important roles of TLR4 and ER signaling in the insulin‐desensitizing effects of the SARS‐CoV‐2 spike protein and COVID‐19 vaccine.

### 2.3 mRNA COVID‐19 Vaccine Boosters Aggravate Insulin Resistance in Patients With Diabetes

**FIGURE 5:**
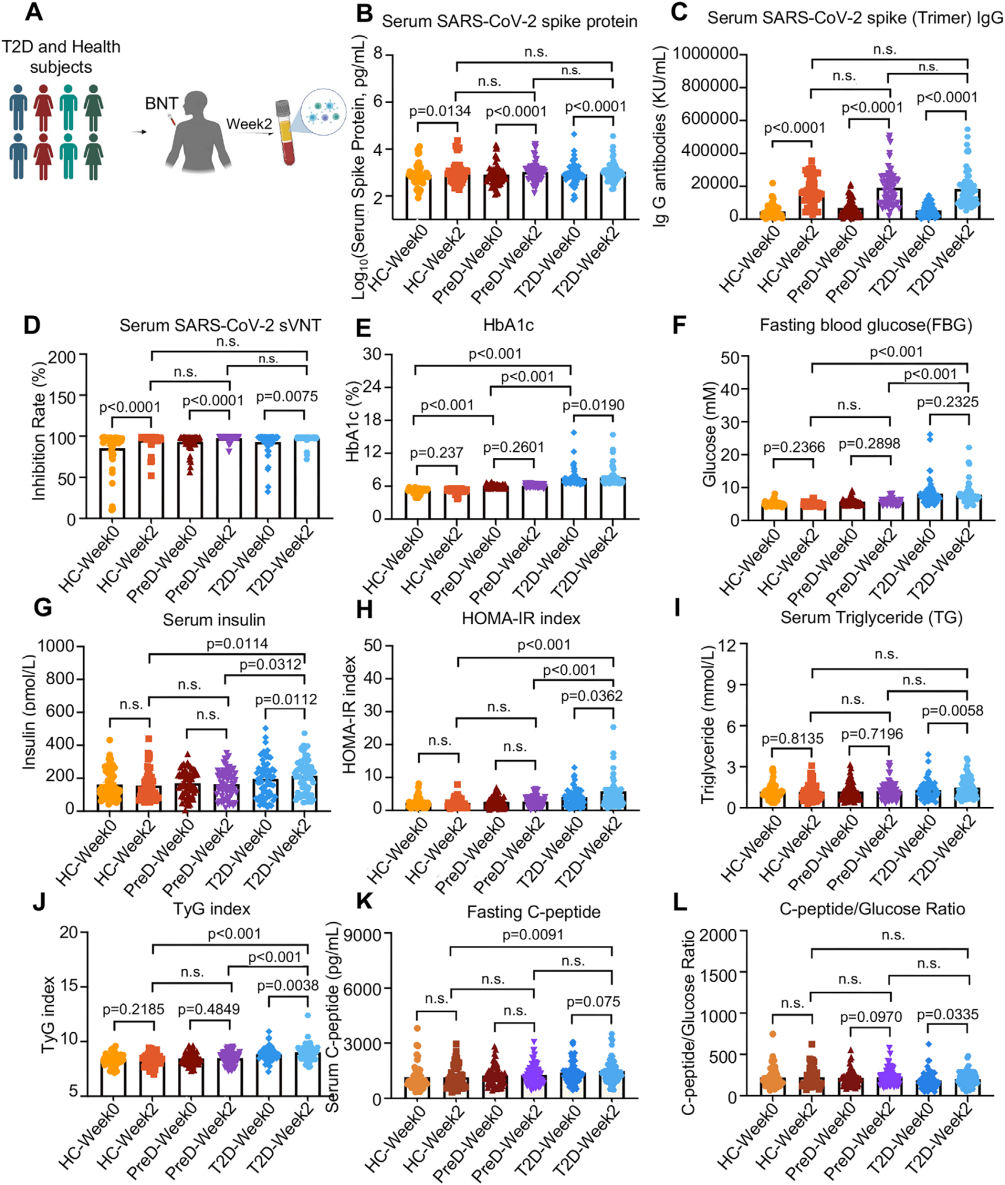
mRNA COVID‐19 vaccine booster impairs insulin sensitivity in patients with diabetes. Experimental design for mRNA COVID‐19 booster vaccination in healthy controls and pre‐diabetic and diabetic patients. (B–D) SARS‐CoV‐2 spike protein, SARS‐CoV‐2 spike (trimer) IgG, and SARS‐CoV‐2 sVNT in serum samples from healthy controls (HC), patients with pre‐diabetes (PreD), and patients with type 2 diabetes (T2D) following mRNA COVID‐19 vaccination (determined by two‐tailed paired *t*‐tests between week 0 and week 2 in each group, determined by one‐way ANOVA between different groups at same time point). (E–L) HbA1c, fasting blood glucose (FBG), insulin, homeostatic model assessment for insulin resistance (HOMA‐IR), triglyceride (TG), triglyceride‐glucose (TyG), C‐peptide, and C‐peptide‐to‐glucose ratio index levels in serum samples from patients with diabetes following vaccination of mRNA COVID‐19 vaccine (determined by two‐tailed paired *t*‐tests between week 0 and week 2 in each group, determined by one‐way ANOVA between different groups at the same time point).

Based on the results in mice, we conducted a prospective cohort study to determine the effects of COVID‐19 vaccine boosters on healthy controls, patients with pre‐diabetes, and patients with diabetes (T2D only) (Figure 5A). Between June 1, 2023, and October 31, 2023, we recruited 180 participants (50 healthy controls, 64 patients with pre‐diabetes, and 66 patients with diabetes) who had received two or more doses of the mRNA COVID‐19 vaccine (BNT162b2). The information of the participants, including age, sex, body mass index (BMI), vaccination doses and interval, COVID infection records, and anti‐diabetic medication records, is shown in Table 1. Human volunteers were recruited to determine their immune responses and metabolic indicators, including glucose and lipid metabolism indices, before and 2 weeks after the booster shot of the COVID‐19 mRNA vaccine (Table S2). As hypothesized, we found that the levels of the SARS‐CoV‐2 spike protein, IgG antibodies against the SARS‐CoV‐2 trimeric spike protein, and the neutralizing ability of the SARS‐CoV‐2 protein significantly increased after vaccination with a COVID‐19 mRNA booster in healthy controls, patients with pre‐diabetes, and patients with diabetes (all *p* < 0.001, Figure 5B–D and Table S2). No significant differences were found in immune response indices among healthy controls and patients with pre‐diabetes or diabetes (Figure 5B–D and Table S2), suggesting that patients with pre‐diabetes and diabetes exhibited similar immune responses to the COVID‐19 mRNA booster as healthy controls.

**TABLE 1:** Demographics and clinical characteristics of participants.

Characteristics	Healthy controls (HC)	Pre‐diabetes (PreD)	Type 2 diabetes (T2D)
*n*	50	64	66
Sex (*n*, ratio%)	Male 31 (62.0%)	Male 36 (56.26%)	Male 39 (59.09%)
	Female 19 (38.0%)	Female 28 (43.74%)	Female 27 (40.91%)
Age (years), (median, IQR)	52 (11)	56 (10)	55 (14)
Weight (kg), (median, IQR)	61 (22)	70 (23)	76 (25)
Height (cm), (median, IQR)	165 (12)	166 (13)	168 (13)
BMI (kg/m^2^), (median, IQR)	23 (4)	25 (6)	28 (8)
Days after the first shot of the COVID‐19 vaccine (median, IQR)	776 (97)	801 (100)	791 (103)
Vaccination times (*n*, ratio%)			
0	3 (6.00%), excluded	0 (0.00%)	1 (1.52%), excluded
1	0 (0.00%)	0 (0.00%)	0 (0.00%)
2	6 (12.0%)	2 (3.125%)	6 (9.09%)
3	28 (56.0%)	29 (45.31%)	25 (37.88%)
4	11 (22.00%)	27 (42.19%)	32 (48.48%)
5	2 (4.00%)	6 (9.375%)	2 (3.03%)
COVID‐19 infection history (*n*, ratio%)	32 (64.0%)	40 (62.5%)	47 (71.21%)
Medication history (*n*, ratio%)			
Metformin	2 (4.00%)	32 (50.00%)	50 (75.76%)
Insulin	0 (0.00%)	2 (3.125%)	13 (19.70%)
Glizlazide	0 (0.00%)	9 (14.06%)	17 (25.76%)
Anti‐hypertensive medication	3 (6.00%)	26 (40.63%)	42 (63.64%)
Cholesterol medication	1 (2.00%)	27 (42.19%)	45 (68.18%)

**FIGURE 6:**
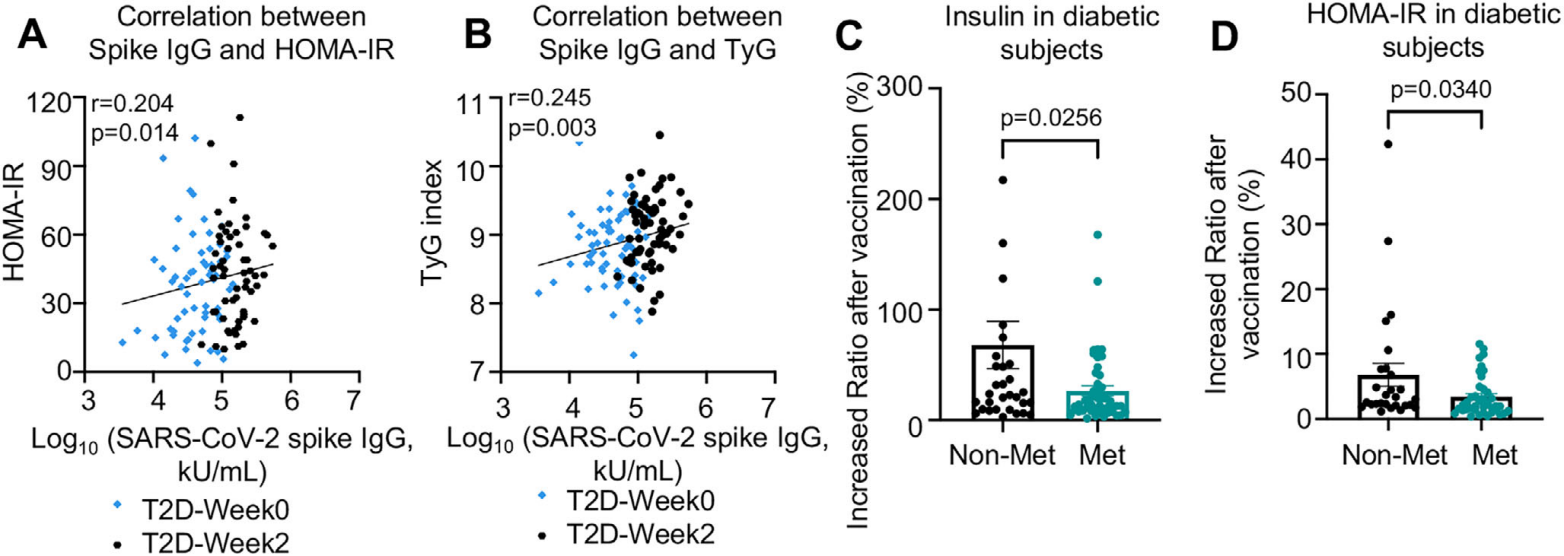
Metformin use is linked with improved insulin sensitivity in patients with diabetes receiving mRNA COVID‐19 vaccine booster. (A and B) Spearman's correlation between serum SARS‐CoV‐2 spike (trimer) IgG, and HOMA‐IR and TyG in patients with diabetes (T2D) following mRNA COVID‐19 vaccination (statistical significance determined by two‐tailed *t*‐tests). (C and D) Insulin and HOMA‐IR indices in the T2D group after COVID‐19 vaccination with/without metformin medication, as revealed by proportional changes from baseline (week 0) to week 2 (statistical significance determined by two‐tailed unpaired *t*‐tests).

However, in contrast to the immune responses to COVID‐19 mRNA boosters observed in all participants, we found exacerbated risks of glucose intolerance and insulin resistance after booster shots of the COVID‐19 mRNA vaccine in patients with diabetes. Specifically, patients with diabetes had a substantial elevation of HbA1c levels, homeostatic model assessment for insulin resistance (HOMA‐IR), TGs, and the triglyceride‐glucose (TyG) index (all *p* < 0.02, Figure 5E–L and Table S2). Although no significant change in FBG was found in human participants after the COVID‐19 mRNA booster shots (Figure 5F and Table S2), 43 of 65 (66.1%) patients with diabetes had impaired insulin sensitivity according to the HOMA‐IR and an increased risk of metabolic dysfunction according to the TyG index. Moreover, correlation analysis revealed that the HOMA‐IR and TyG indices were positively correlated with the levels of SARS‐CoV‐2 trimeric spike IgG in subjects with diabetes (*r* = 0.204 and 0.245, respectively; both *p* < 0.05; Figure 6A,B and Table S2). These results suggest that booster immunization with the COVID‐19 mRNA vaccine is linked to insulin resistance only in patients with T2D.

Considering that heterogeneity in vaccine dosing may influence outcomes, including immune responses and metabolic results, we performed a subgroup analysis to compare the outcomes between participants receiving different numbers of vaccine doses. In line with our findings, insulin resistance was aggravated in subjects who received more than two doses, and only six participants received ≤ 2 doses in this study (Figure S4A–I). Subsequently, we investigated the correlation between immune responses and metabolic health indices in patients with T2D receiving more than two‐dose booster regimens of COVID‐19 vaccines. Immune response‐related factors including the levels of the SARS‐CoV‐2 spike protein, IgG antibodies against the SARS‐CoV‐2 trimeric spike protein, and the neutralizing ability of the SARS‐CoV‐2 protein were positively associated with TGs and insulin (*r* > 0.15, *p* < 0.05; Figure S5).

### 2.4 Metformin Alleviates Insulin Resistance in mRNA COVID‐19 Vaccine‐Treated Mice

Metformin, a first‐line anti‐diabetic drug, ameliorates the severity and mortality risk of COVID‐19 [15] and reduces the incidence of post‐acute COVID‐19 syndrome (PACS) [16]. We assessed the prevalence of metformin use (Table 1) and its relevance to insulin resistance in patients with diabetes before and after receiving the COVID‐19 mRNA booster vaccine. Interestingly, we found that insulin resistance was less affected by the COVID‐19 mRNA boosters in patients with diabetes who were taking metformin during the study (*p* < 0.05, Figure 6C,D). This finding suggests that metformin may be advantageous for patients with diabetes by managing blood glucose levels and mitigating insulin resistance after administration of COVID‐19 mRNA boosters.

**FIGURE 7:**
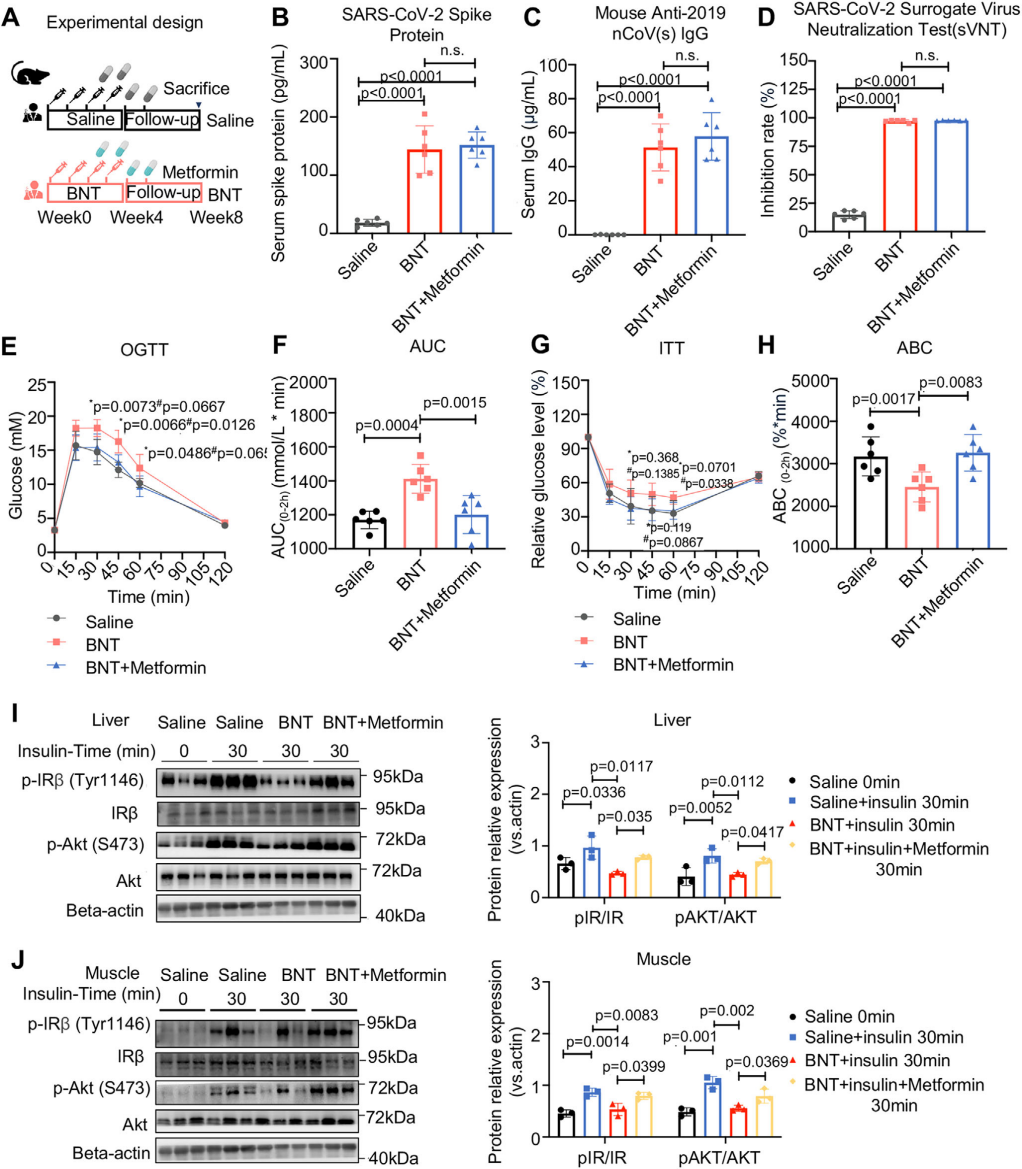
Metformin alleviates insulin resistance induced by COVID‐19 vaccine in mice. (A) Experimental design for the treatment of mRNA COVID‐19 vaccine in combination with metformin in healthy mice. (B–D) SARS‐CoV‐2 spike protein, SARS‐CoV‐2 spike (trimer) IgG, and SARS‐CoV‐2 sVNT in serum samples of normal mice following treatment with the mRNA COVID‐19 vaccine (4.5 µg/kg) and metformin (300 mg/kg) (*n* = 6/group; statistical significance determined by two‐tailed one‐way ANOVA test). (E and F) OGTT and AUC indices in normal mice following treatment of mRNA COVID‐19 vaccine (4.5 µg/kg) and metformin (300 mg/kg) (*n* = 6/group; OGTT determined by two‐way ANOVA, AUC determined by two‐tailed *t*‐tests). *Comparisons between the BNT and the saline group. ^#^Comparisons between the BNT group and metformin treatment (2 mg/kg). (G and H) ITT and ABC indices in mice following treatment of mRNA COVID‐19 vaccine (4.5 µg/kg) and metformin (300 mg/kg) (*n* = 6/group; ITT determined by two‐way ANOVA, ABC determined by two‐tailed t‐tests). *Comparisons between BNT group and saline group. ^#^Comparisons between the BNT group and metformin treatment. (I and J) Western blot (and quantification) demonstrated the effect of mRNA COVID‐19 vaccine (4.5 µg/kg) and metformin (300 mg/kg) on IR and AKT phosphorylation levels in liver and muscle lysates (*n* = 6/group; statistical significance determined by two‐tailed one‐way ANOVA test).

Considering that the beneficial effects of metformin on insulin resistance in the context of COVID‐19 remain unclear, we investigated the effect of metformin on variations in insulin sensitivity induced by the COVID‐19 vaccine in mice (Figure 7A). Notably, metformin at a clinically relevant dose (300 mg/kg) did not affect the protective effects of the COVID‐19 vaccine against SARS‐CoV‐2, as demonstrated by the lack of significant changes in the serum levels of SARS‐CoV‐2 spike protein, spike protein IgG, and SARS‐CoV‐2 virus neutralization test (sVNT) in mice receiving COVID‐19 mRNA vaccines (Figure 7B–D). We showed that metformin alleviated insulin resistance in mice treated with four doses of COVID‐19 vaccine, as revealed by improved OGTT and ITT indices (*p* < 0.05, Figure 7E–H) and improved insulin signaling (*p* < 0.05, Figure 7I–J). To further validate the effects of the SARS‐CoV‐2 spike protein on insulin sensitivity and the metabolic benefits of metformin, we employed a well‐established mouse model of diabetes (*db*/*db* mice; Figure S6A). Consistent with our previous findings, two doses of BNT162b2 significantly exacerbated glucose intolerance and insulin resistance in *db/db* mice, whereas metformin effectively alleviated impaired glucose intolerance and insulin resistance but did not affect the serum levels of SARS‐CoV‐2 spike protein (*p* < 0.001 in all cases, Figure S6B–F). These results suggest that metformin can be used as an adjuvant therapy to maintain glucose control during COVID‐19 vaccination without significantly impeding immunogenic responses.

**FIGURE 8:**
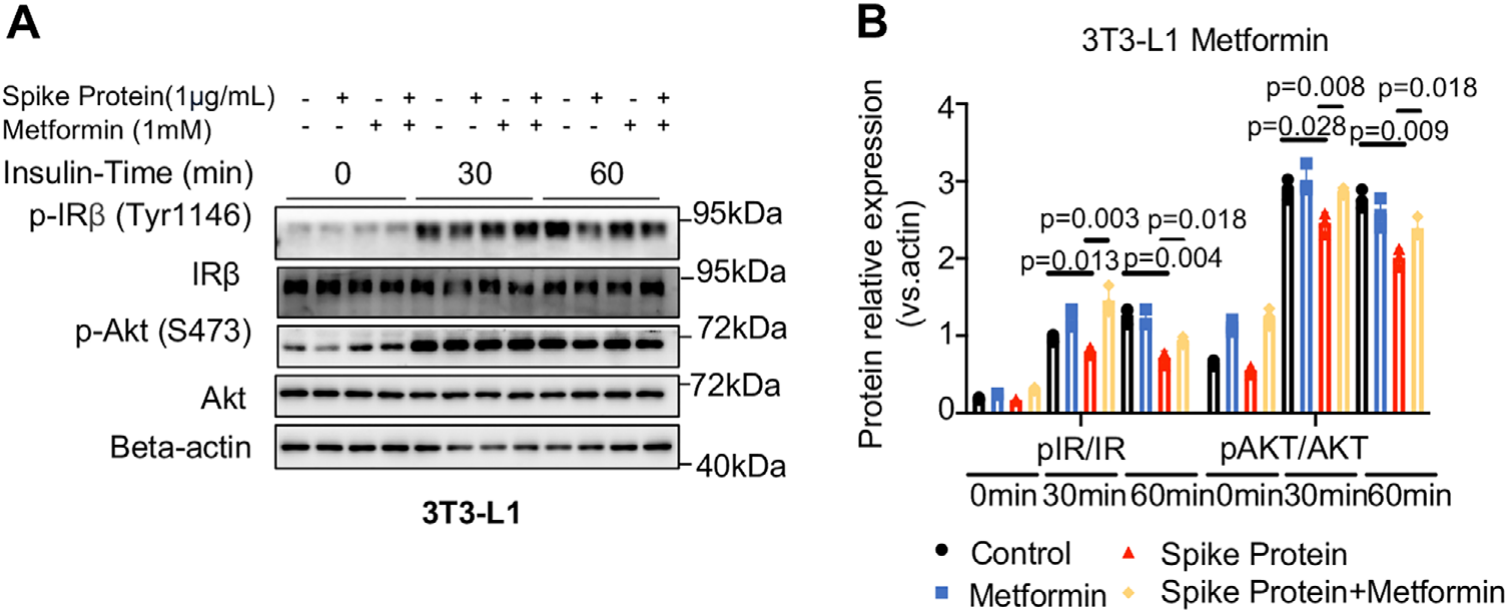
Metformin rescues impaired insulin signaling induced by SARS‐CoV‐2 spike protein in 3T3‐L1 cells. (A and B) Western blot (and quantification) demonstrated the effect of spike protein (1 µg/mL) and metformin (1 mM) on IR and AKT phosphorylation (*n* = 3/group) in 3T3‐L1 cells (statistical significance determined by two‐tailed one‐way ANOVA test).

In line with the in vivo findings, we validated the beneficial action of metformin on insulin sensitivity in 3T3‐L1 cells, as weakened insulin signaling induced by the SARS‐CoV‐2 spike protein was also significantly improved by metformin treatment (*p* < 0.05, Figure 8A,B). Taken together, these results suggest that the SARS‐Cov‐2 spike protein, a target of the COVID‐19 vaccine, disrupts insulin signaling only in patients with T2D, whereas metformin attenuates the COVID‐19 vaccine booster‐induced changes in insulin sensitivity. The results from this study do not negate the clinical benefits of the COVID‐19 vaccine but highlight the need for extra monitoring of glycemic control in patients with diabetes who are going to receive COVID‐19 vaccine boosters.

## 3 Discussion

The rapid development and widespread distribution of SARS‐CoV‐2 vaccines have played a crucial role in mitigating the global impact of the COVID‐19 pandemic. Although these vaccines have proven highly effective in preventing severe COVID‐19 infection and reducing transmission rates, the long‐term effects of COVID‐19 vaccines on patients with underlying health conditions are not well understood [11]. In this study, we showed that COVID‐19 boosters (≥4) significantly impaired insulin signaling in healthy mice, accompanied by an elevation of the SARS‐CoV‐2 spike protein and IgG antibodies against the SARS‐CoV‐2 spike protein, resulting in impaired glucose control and insulin signaling. Mechanistically, we demonstrated that the SARS‐CoV‐2 spike protein disrupts insulin signaling by binding to a series of receptors, including ACE2, TLR4, and ER. Moreover, booster COVID‐19 vaccines specifically weakened insulin sensitivity in patients with diabetes, providing a causal link between COVID‐19 vaccines and variations in insulin signaling. Several studies have assessed the impact of COVID‐19 vaccines on glucose metabolism and insulin sensitivity [4, 17]. These studies found no significant changes in glucose tolerance, as measured by FBG levels, following a single vaccine dose in the general population. In contrast, we showed that booster shots of the COVID‐19 vaccine altered insulin sensitivity and increased the risk of diabetic complications in patients with diabetes, as measured using biological indices of insulin sensitivity.

Heterogeneity in vaccine dosing among the study participants may affect the interpretation of insulin resistance findings. Only six participants received two or more doses, therefore, the small sample size limited a robust comparison. The observed increase in insulin resistance with more than two doses suggests a possible dose–response relationship; however, this is difficult to confirm without balanced dosing groups. Variability in immune responses and potential confounding factors, such as differences in age or health status, further complicate the analysis. Therefore, our future research will aim to achieve a more even distribution of dosing levels to better understand the metabolic effects of different vaccine regimens.

The enrichment analysis highlighted significant alterations in several key signaling pathways, including NF‐κB, AMPK, MAPK, mTOR, and PI3K‐Akt. These findings are crucial for understanding the mechanisms by which COVID‐19 vaccines affect glucose tolerance. Alterations in NF‐κB and MAPK pathways suggest that vaccination impacts energy metabolism and cellular stress responses [18, 19]. The AMPK, mTOR, and PI3K‐Akt pathways are well‐known regulators of cell growth, proliferation, and glucose metabolism, which further supports the link between vaccination and glucose control [20, 21]. The observed alterations in these signaling pathways provide insights into how COVID‐19 vaccines might influence metabolic processes, including glucose metabolism.

Interestingly, we noticed that the use of metformin, a common anti‐diabetic medication, was associated with reduced insulin resistance in patients with diabetes following vaccination with COVID‐19 boosters. Metformin is associated with improved clinical outcomes of COVID‐19 and a reduced risk of developing PACS‐related symptoms [16]. AMP‐activated protein kinase (AMPK) regulates cellular energy and improves insulin sensitivity [22]. Metformin, a potent AMPK activator, was able to counteract insulin signaling disruptions caused by the mRNA COVID‐19 vaccine in mice without affecting the levels of the SARS‐CoV‐2 spike protein. Our data indicated that metformin mitigates the adverse effects of the spike proteins through AMPK activation. Further studies using AMPK inhibitors and shRNA could help confirm this mechanism. In addition to metformin, therapeutic strategies including glucagon‐like peptide‐1 (GLP‐1) agonists and sodium‐glucose co‐transporter‐2 (SGLT‐2) inhibitors help manage insulin sensitivity and support overall metabolic health [23]. The use of such drugs could be advantageous for patients experiencing altered insulin sensitivity post‐vaccination, as these therapeutics help maintain glucose control without directly affecting the insulin pathway. Studies have shown that PACS is linked to the persistence of the SARS‐CoV‐2 spike protein and increased IgG antibodies against the SARS‐CoV‐2 spike protein. Impaired insulin signaling was associated with PACS in patients who also have an increased risk of diabetes and diabetes‐related complications [24]. Our study suggests that metformin offers metabolic benefits not only to patients with diabetes preparing for SARS‐CoV‐2 vaccination, but also to individuals infected with COVID‐19 or suffering from PACS.

Recent studies have also revealed an association between COVID‐19 and the risk of T2D. A large, retrospective cohort study found that patients with a history of COVID‐19 had a significantly higher risk of developing T2D within 1 year of infection than the general population [25]. Studies have reported an increased risk of new‐onset diabetes in individuals who have recovered from COVID‐19 [26, 27]. Viruses often manipulate the host organisms’ metabolic pathways to enhance viral replication and survival. By blocking insulin signaling, viruses can affect glucose tolerance and potentially suppress immune defenses, thereby aiding viral replication. For instance, hepatitis C virus (HCV) induces insulin resistance via oxidative stress and inflammatory cytokines, whereas human immunodeficiency virus (HIV) and HIV therapy are linked to insulin resistance and metabolic disturbances [28, 29].

In addition to our findings, there are several potential mechanisms through which COVID‐19 boosters may increase the risk of T2D development in patients with pre‐diabetes. First, SARS‐CoV‐2 spike protein production guided by COVID‐19 boosters may contribute to insulin resistance and impaired glucose metabolism [3]. The SARS‐CoV‐2 spike protein may persist several months after COVID‐19 vaccination, affecting the development of T2D in susceptible individuals [30]. In addition to ACE2, the SARS‐CoV‐2 spike protein induced by COVID‐19 boosters binds to TLR4 and ER. Both TLR4 and ER have been implicated in the dysregulation of metabolic homeostasis [31, 32]. Briefly, TLR4 activation by either lipopolysaccharides or fatty acids leads to metabolic inflammation and insulin resistance via NF‐κB signaling axis, while estrogen deficiency or impaired estrogen signaling is associated with insulin resistance and dysregulation of metabolic homeostasis. Estrogen exerts anti‐inflammatory effects by suppressing pro‐inflammatory cytokine production via the TLR4 pathway [33]. Studies have suggested that ER modulates downstream TLR4 signaling molecules, such as MyD88 and TRIF, thereby dampening the inflammatory response [34]. This crosstalk may reduce TLR4 signaling when the ER is activated. Further studies are necessary to explore these mechanisms in the context of SARS‐CoV‐2 spike protein interactions.

Second, the SARS‐CoV‐2 spike protein is known to cause systemic immune responses and the production of IgG antibodies against the SARS‐CoV‐2 spike protein by the host, which may affect the function of insulin signaling pathways and lead to insulin resistance [35, 36]. We also noted that insulin sensitivity and TG levels were affected by COVID‐19 boosters in patients with diabetes but not in healthy controls or patients with pre‐diabetes; these findings suggest that individuals with impaired glucose tolerance should pay close attention to their blood glucose homeostasis after COVID‐19 vaccination [37]. A longitudinal study design can help better monitor glucose control and complication risks among patients with glucose intolerance receiving the COVID‐19 vaccine, and provide clinical evidence for the metabolic benefits of metformin in patients with diabetes. Further understanding of the molecular mechanisms of action of metformin on spike protein‐induced glucose intolerance could provide valuable insights into managing metabolic disturbances in patients with diabetes, particularly in the context of COVID‐19 and its vaccines.

In addition to the concerns related to COVID‐19 and vaccination revealed in this study, diabetes is associated with several chronic complications that require ongoing management and attention. High blood glucose levels can damage retinal blood vessels and lead to diabetic retinopathy [38]. Ophthalmological examinations are recommended for early detection and management of patients with diabetes who have recovered from COVID‐19. Moreover, complications such as diabetic neuropathy and peripheral vascular disease require clinical care, including regular health checking and glucose control [39, 40]. While COVID‐19 vaccines are essential for preventing severe disease, understanding and mitigating their potential effects on insulin sensitivity are vital for optimizing patient outcomes. Additionally, addressing diabetes complications through regular monitoring and preventative care is crucial for improving the quality of life of individuals with diabetes.

## 4 Materials and Methods

The experiments were not randomized, and the investigators were not blinded to the allocation during the experiments and outcome assessment.

### 4.1 Reagents and Resources

Reagents and resource details are provided in Table S3.

### 4.2 Human Participants

A total of age‐matched 180 participants receiving the BioNTech (BNT) mRNA COVID‐19 vaccination (66 in the diabetes mellitus group, 64 in the pre‐diabetes mellitus group, and 50 in the healthy control group) were recruited. The participants were observed over a 2‐week period for changes in insulin sensitivity after BNT vaccination to determine the effects of spike protein on insulin sensitivity. Written informed consent for publication of their details was obtained from the study participant.

### 4.3 Animals

Mouse experiments were performed according to the regulations of the animal ordinance of the Department of Health, Hong Kong SAR, China. Male BALB/c mice (6–8 weeks old and 20–25 g) purchased from the Laboratory Animal Services Center of the Chinese University of Hong Kong were raised in the Animal Unit of the School of Chinese Medicine at Hong Kong Baptist University. Male *db*/*db* mice (6–8 weeks old and 20–25 g) were purchased from Jiangsu Huachuang Sino Pharma Tech Co., Ltd. The mice were maintained under a 12‐h/12‐h light/dark cycle with a controlled temperature of approximately 25°C and ad libitum to food and water.

In the first experiment, mice were administered the BioNTech COVID‐19 mRNA vaccine by intramuscular injection at a dose of 4.5 µg/kg once per week for 4 weeks, according to the dosage used in humans [9]. In the second study, mice were administered either the BioNTech COVID‐19 mRNA vaccine by intramuscular injection at a dose of 4.5 µg/kg, and co‐administered with a related activator/agonist or inhibitor/antagonist of TLR4, ER, and ACE2. In the third study, mice were administered a BioNTech COVID‐19 mRNA vaccine by intramuscular injection at a dose of 4.5 µg/kg and co‐treated with or without metformin by oral gavage at a dose of 300 mg/kg.

### 4.4 OGTT and Insulin Glucose Tolerance Test (ITT)

The mice were fasted overnight (12 h) before the OGTT and orally administered a glucose solution (2 g/kg). For glucose measurement, blood samples were collected from the caudal vein of each mouse at 0, 15, 30, 45, 60, 90, and 120 min after glucose administration immediately. The area under the concentration‐time curve (AUC) was calculated based on glucose index. For the ITT, mice were fasted for 4 h prior to testing and administered insulin by intraperitoneal administration at a dose of 1 U/kg. Similar to the OGTT, blood samples for glucose measurement were collected from the caudal vein of each mouse at 0, 15, 30, 45, 60, 90, and 120 min. The area above the concentration‐time curve (ABC) was calculated.

### 4.5 Serological Tests

The mice were fasted overnight (12 h) before serum sample collection. Serum samples (200 µL) were collected by orbital bleeding in anesthetized mice. The SARS‐CoV‐2 spike RBD IgG test, sVNT, SARS‐CoV‐2 spike protein ELISA, and TG and insulin levels were measured according to the manufacturer's protocols.

### 4.6 Western Blotting

Frozen mouse tissue samples and harvested cells obtained from experiments mentioned above were lysed in RIPA buffer and normalized to a concentration of 2 mg/mL protein. For western blotting, tissue and cell lysates were each mixed with 5x loading buffer and heated at 98°C for 10 min. Target proteins were detected according to the instructions from BioRad. The blots were then incubated with HRP‐conjugated anti‐rabbit or anti‐mouse IgG and reacted with chemiluminescence. The blots were semi‐quantified using ImageJ software.

### 4.7 Transcriptomics by RNA‐Sequencing

Frozen liver tissues were used to extract RNA using TRIzol reagent following the manufacturer's protocol and quantified using a NanoDrop One Spectrophotometer. The mRNA was then transcribed into cDNA and purified using the QiaQuick PCR extraction kit. The reaction system and program for adaptor ligation were configured to ligate adaptors to cDNAs to amplify the product. The ligation products were amplified using PCR and sequenced using the DNBSEQ sequencing system. Data analysis, including data filtering, RNA identification, gene quantification, differential expression analysis, and gene annotation, was performed according to our previous study [41].

### 4.8 Cell Culture

The 3T3‐L1 (ATCC CL‐173), C2C12 (ATCC CRL‐1772), and HepG2 cells (ATCC HB‐8065) were cultured in Dulbecco's modified Eagle medium (DMEM) with 10% fetal bovine serum (FBS) and 1% penicillin‐streptomycin under 5% CO_2_ at 37°C. The 3T3‐L1 cells were grown overnight (12 h) before treatment. For the glucose uptake assay, 3T3‐L1 cells were serum and glucose starved for 4 h and then incubated with SARS‐CoV‐2 spike protein (1 µg/mL). To study the mechanism of action of the SARS‐CoV‐2 spike protein on insulin signaling, 3T3‐L1 cells were pretreated with or without SARS‐CoV‐2 spike protein and related activators/agonists or inhibitors/antagonists of TLR4, ER, and ACE2 at the indicated dosages and times. All cell lines utilized in this study were authenticated using short tandem repeat (STR) analysis and confirmed to be free of mycoplasma contamination.

### 4.9 Quantitative Real‐Time Polymerase Chain Reaction

Total RNA from mouse liver tissues and 3T3‐L1 cells was isolated using TRIzol reagent. The cDNA was constructed from 0.5 µg of the total RNA by using a PrimeScript RT Master Mix. The primers used are listed in Table S3.

### 4.10 Statistical Analysis

Data are expressed as average and SD values of at least triplicate measurements. *p*‐values were calculated using GraphPad Prism 9, and *p*‐values less than 0.05 are considered statistically significant. Unpaired Student's *t*‐tests or one‐way ANOVA were employed in the analysis settings.

## Author Contributions

Conceptualization: Z.‐X.B., H.L.X.W., and L.X.Z. Methodology: H.L.X.W., L.X.Z., M.Z., and C.Y.L. Investigation: M.Z., G.Y.B., Y.J.Z., S.J.X., H.K.W., J.L.Z., J.Y.L., and H.R.Y. Visualization: M.Z. and L.X.Z. Funding acquisition: Z.X.B. and H.L.X.W. Project administration: Z.‐X.B., H.L.X.W., L.X.Z., and C.Y.L. Supervision: Z.‐X.B., H.L.X.W., and L.X.Z. Writing – original draft: L.X.Z. and M.Z. Writing – review and editing: Z.‐X.B., H.L.X.W., L.X.Z., and S.F.Y. All authors have read and approved the final manuscript.

## Ethics Statement

The study involving humans was approved by the Research Committee on the Use of Human and Animal Subjects in Teaching and Research at Hong Kong Baptist University (REC/22‐23/0277) and was registered in the Chinese Clinical Trial Registry (ChiCTR2300069830). Written informed consent was obtained from all of the participants. The experiments involving mice were approved by the Research Committee on the Use of Human and Animal Subjects in Teaching and Research at the Hong Kong Baptist University (REC/22‐23/0611).

## Conflicts of Interest

The authors declare no conflicts of interest.

## Supporting information




**Supporting Figure S1**: The mRNA COVID‐19 vaccine disrupts insulin signaling in mice.
**Supporting Figure S2**: Effect of inactivated COVID‐19 vaccine on glucose tolerance in mice.
**Supporting Figure S3**: SARS‐CoV‐2 spike protein impairs insulin signaling via multiple signaling pathways.
**Supporting Figure S4**: Vaccination with the mRNA COVID‐19 booster impairs insulin sensitivity in patients with type 2 diabetes.
**Supporting Figure S5**: Correlation analysis between immune responses and metabolic health indices in patients with type 2 diabetes.
**Supporting Figure S6**: Metformin alleviates insulin resistance induced by COVID‐19 vaccination in *db/db* mice.
**Supporting Table S2**: Clinical characteristics in healthy controls and patients with pre‐diabetes and diabetes.
**Supporting Table S3**: Reagents and resources used in this study.

Table S1 mRNA gene expression after COVID‐19 vaccination in mice and enrichment analysis (standalone Excel file).

## Data Availability

Further information and requests for basic and clinical resources can be directed to Lixiang Zhai (lxzhai@hkbu.edu.hk) and Zhao‐xiang Bian (bzxiang@hkbu.edu.hk). No new experimental materials were generated in this study.
