# Supplementary material for: SARS‐CoV‐2 Spike Protein as a Target of the COVID‐19 Vaccine Disrupts Insulin Signaling in Type 2 Diabetes
Source: MedComm (2020). 2025 Nov 2;6(11):e70469. doi: 10.1002/mco2.70469 (PMC12580406; doi:10.1002/mco2.70469)
Supplement: Supplementary file 1 — Supporting Figure S1: The mRNA COVID‐19 vaccine disrupts insulin signaling in mice. Supporting Figure S2: Effect of inactivated COVID‐19 vaccine on glucose tolerance in mice. Supporting Figure S3: SARS‐CoV‐2 spike protein impairs insulin signaling via multiple signaling pathways. Supporting Figure S4: Vaccination with the mRNA COVID‐19 booster impairs insulin sensitivity in patients with type 2 diabetes. Supporting Figure S5: Correlation analysis between immune responses and metabolic health indices in patients with type 2 diabetes. Supporting Figure S6: Metformin alleviates insulin resistance induced by COVID‐19 vaccination in db/db mice. Supporting Table S2: Clinical characteristics in healthy controls and patients with pre‐diabetes and diabetes. Supporting Table S3: Reagents and resources used in this study. [file MCO2-6-e70469-s001.docx]

**Supplemental Information**

**SARS-CoV-2 spike protein as a target of the COVID-19 vaccine disrupts insulin signaling in type 2 diabetes**

Lixiang Zhai^1, 2, #^, Min Zhuang^1, 2, #^, Hoi Ki Wong^2, #^, Chengyuan Lin^1^, Haoran Ying^1^, Jialing Zhang^1, 2^, Gengyu Bao^1^, Yijing Zhang^2^, Shujun Xu^1, 2^, Jingyuan Luo^1, 2^, Shuofeng Yuan^3^, Hoi Leong Xavier Wong^2,^ *, Zhao-xiang Bian^1, 2,^ *

^1^ Centre for Chinese Herbal Medicine Drug Development, Hong Kong Baptist University, Hong Kong SAR, China

^2^ School of Chinese Medicine, Hong Kong Baptist University, Hong Kong SAR, China

^3^ Department of Microbiology, Li Ka Shing Faculty of Medicine, The University of Hong Kong, Hong Kong SAR, China

^#^ These authors contributed equally

**Supplemental Figures**

Figure S1. The mRNA COVID-19 vaccine disrupts insulin signaling in mice.

Figure S2. Effect of inactivated COVID-19 vaccine on glucose tolerance in mice.

Figure S3. SARS-CoV-2 spike protein impairs insulin signaling via multiple signaling pathways.

Figure S4. Vaccination with the mRNA COVID-19 booster impairs insulin sensitivity in patients with type 2 diabetes.

Figure S5. Correlation analysis between immune responses and metabolic health indices in patients with type 2 diabetes.

Figure S6. Metformin alleviates insulin resistance induced by COVID-19 vaccination in *db/db* mice.

**Supplementary Tables**

Table S1 mRNA gene expression after COVID-19 vaccination in mice and enrichment analysis (standalone Excel file).

Table S2 Clinical characteristics in healthy controls and patients with pre-diabetes and diabetes.

Table S3 Reagents and resources used in this study.


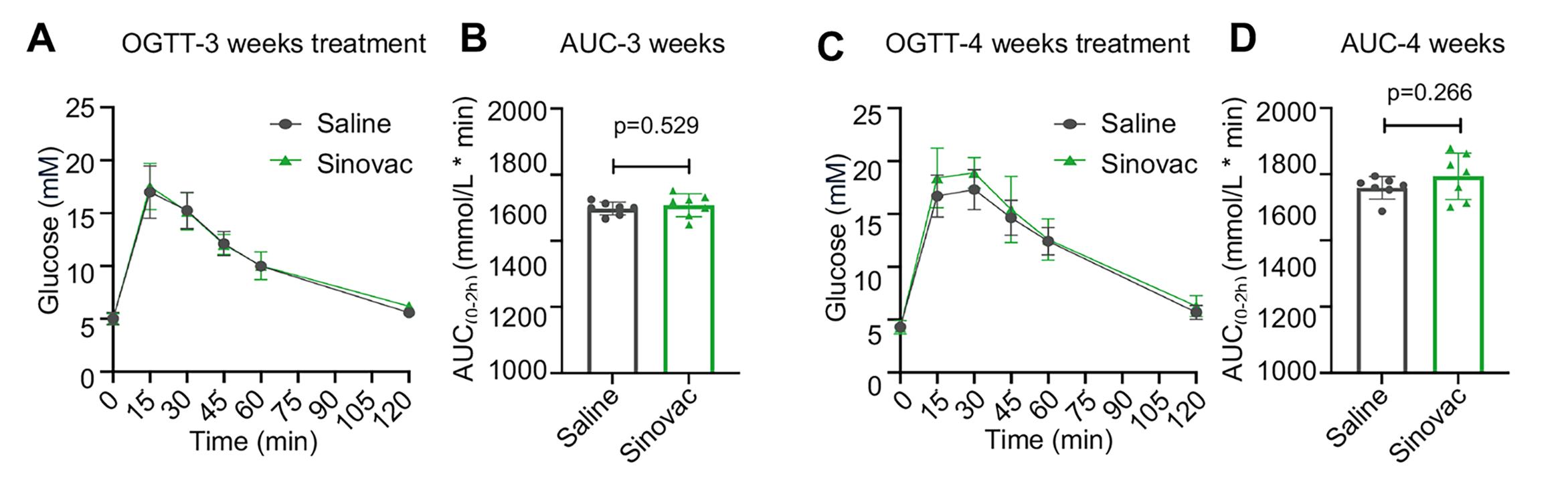


**Figure S1. mRNA COVID-19 vaccine disrupts insulin signaling in mice.** (A-D) OGTT and AUC (area under curve) indexes in normal mice following treatment of BNT mRNA COVID-19 vaccine (4.5 μg/kg) for 2 and 3 weeks (n=7 per group) (OGTT determined by two-way ANOVA, AUC determined by two-tailed t-tests).


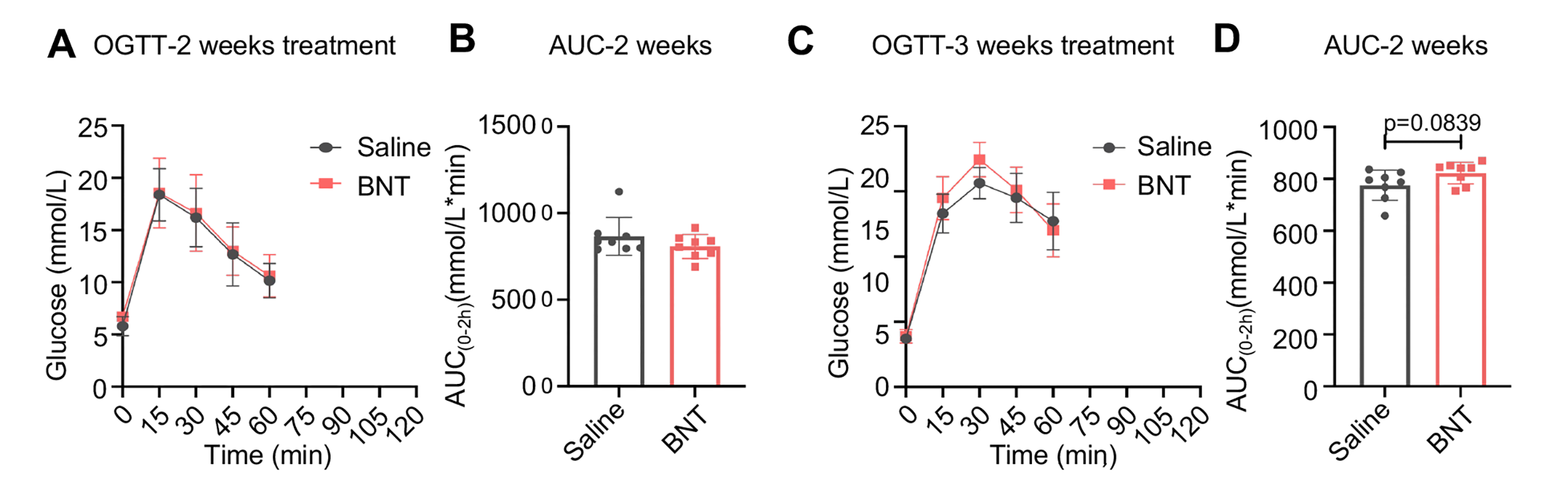


**Figure S2. Effects of inactivated COVID-19 vaccine on glucose tolerance in mice**. **(A-D)** OGTT and AUC indexes in normal mice following treatment of Sinovac COVID-19 vaccine (60SU/kg) for 3 weeks and 4 weeks (n=8 per group) (OGTT determined by two-way ANOVA, AUC determined by two-tailed t-tests).


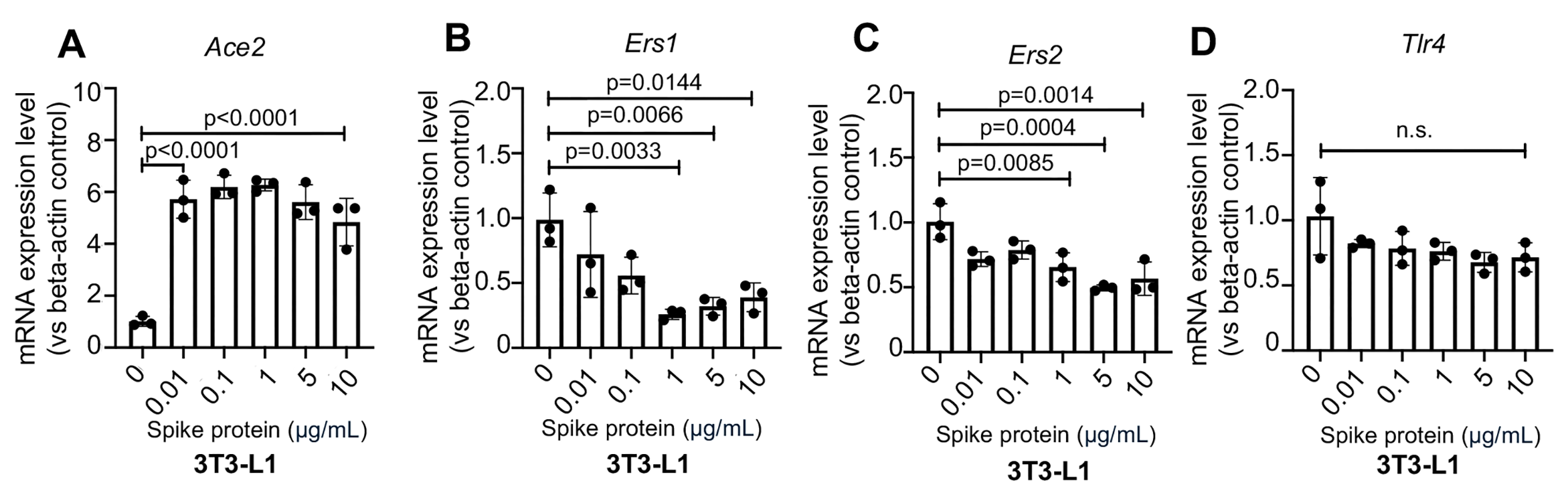


**Figure S3. SARS-CoV-2 spike protein impairs insulin signaling in 3T3-L1 cells. (A-D)** Relative mRNA expression levels of *Ace2*, *Ers1*, *Ers2*, and *Tlr4* in 3T3-L1 cells following treatment with SARS-CoV-2 spike protein (0.01–10 μg/mL) at the indicated dosage (n=3/group; statistical significance determined by two-tailed one-way ANOVA test).

**
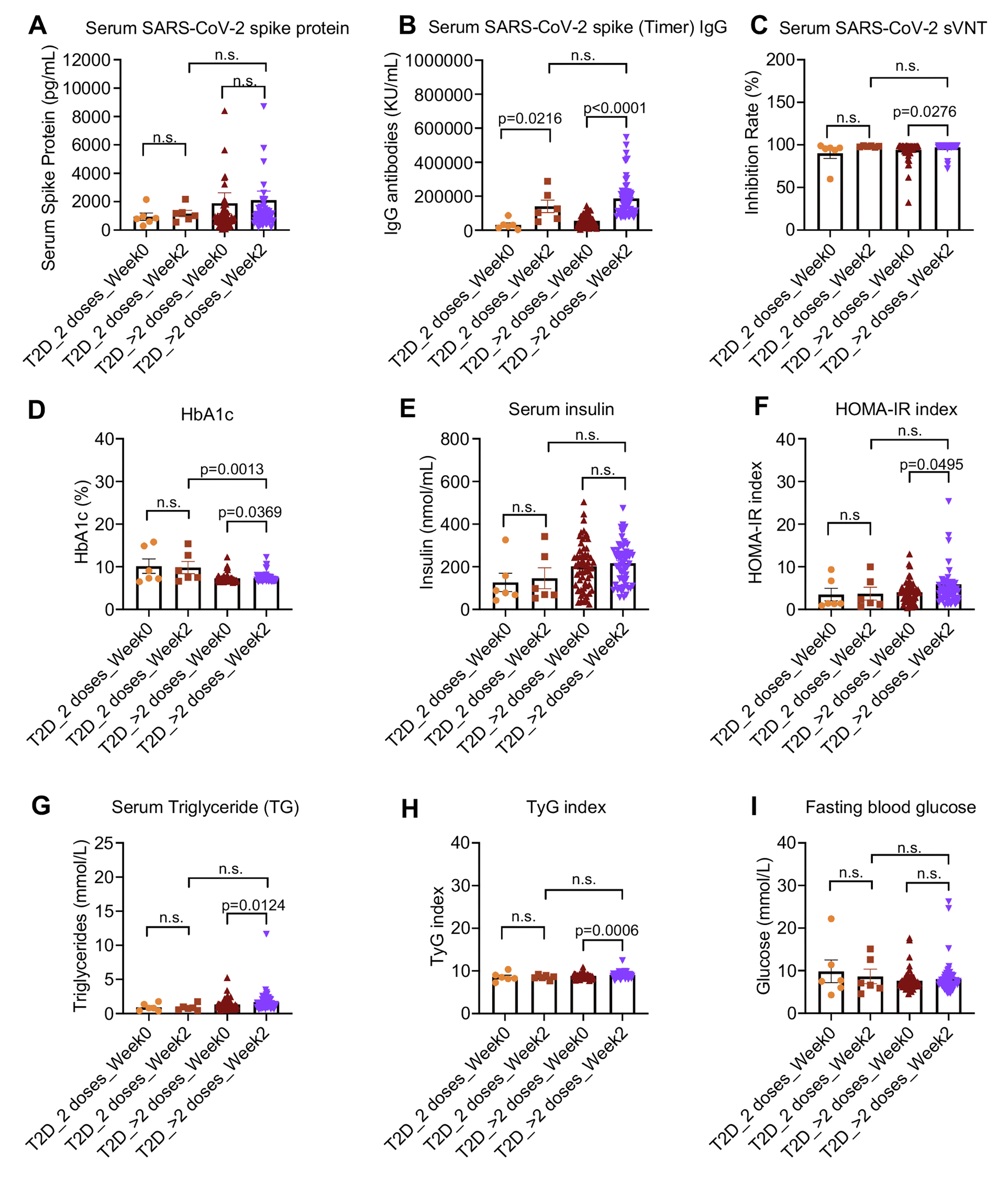
**

**Figure S4. Effects of mRNA COVID-19 vaccine booster on insulin sensitivity in patients with T2D receiving different doses.** **(A-C)** SARS-CoV-2 spike protein, SARS-CoV-2 spike (Trimer) IgG, and SARS-CoV-2 sVNT in serum samples from subjects with T2D following 2 dosages and more than 2 dosages vaccination of mRNA COVID-19 vaccine (determined by two-tailed paired t-tests between week 0 and week 2 in each group, determined by one-way ANOVA between different groups at same time point). **(D-I)** HbA1c, FBG, insulin, HOMA-IR, TG, and TyG index in serum samples from subjects with T2D following 2 dosages and more than 2 dosages vaccination of mRNA COVID-19 vaccine (determined by two-tailed paired t-tests between week 0 and week 2 in each group, determined by one-way ANOVA between different groups at same time point).

**
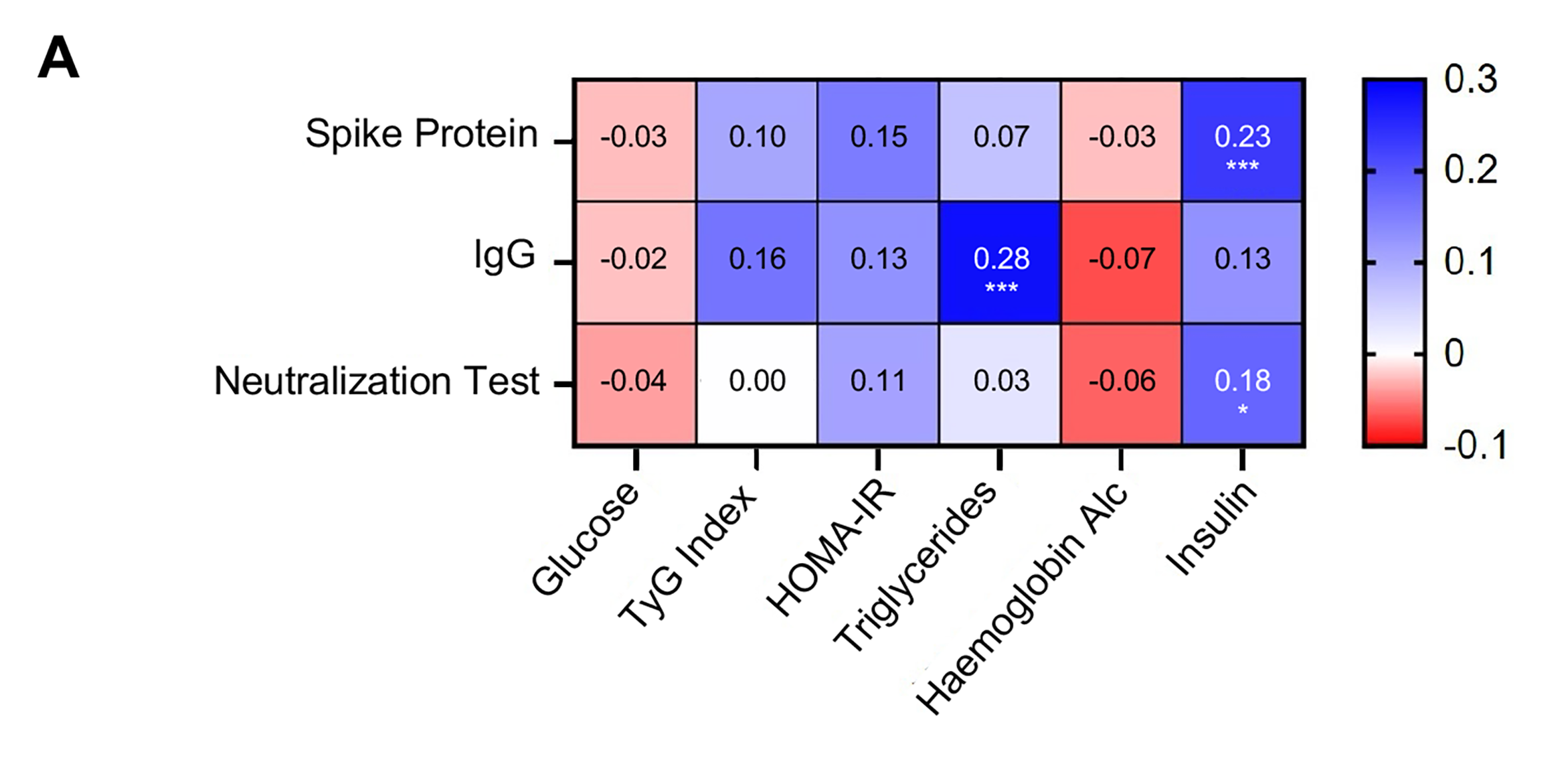
**

**Figure S5. Correlation between immune responses and metabolic indicators in patients with T2D after COVID-19 vaccination.** **(A)** Nonparametric Spearman correlation analysis between serum SARS- Cov2 Spike Protein, Spike Trimer IgG, and SARS-Cov-2 sVNT and with Glucose, TyG Index, HOMA-IR, TG, HbAlc, and insulin in subjects with diabetes (T2D) following more than 2 dosages of mRNA COVID-19 vaccine. *p < 0.05, ** p < 0.01, *** p < 0.001
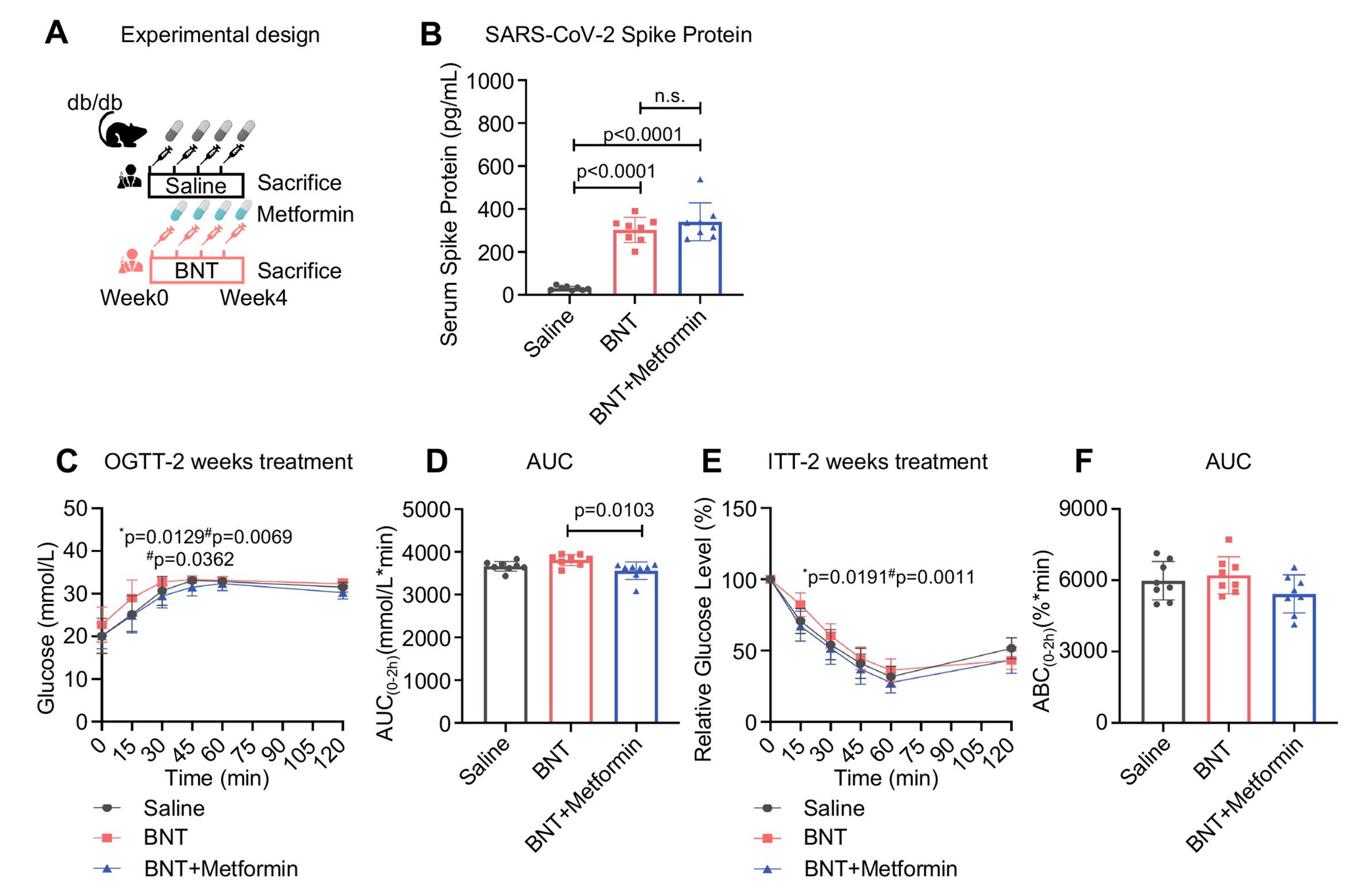


**Figure S6. Metformin alleviates insulin resistance induced by COVID-19 vaccine in *db/db* mice. (A)** Experimental design for treatment of mRNA COVID-19 vaccine in combination with metformin in *db/db* mice. **(B)** SARS-CoV-2 spike protein in serum samples from *db/db* mice following treatment of mRNA COVID-19 vaccine (4.5 μg/kg) and metformin (300mg/kg) (n=8 per group) (determined by two-tailed one-way ANOVA test). **(C-D)** OGTT and AUC indexes in *db/db* mice following treatment of mRNA COVID-19 vaccine (4.5 μg/kg) and metformin (300mg/kg) (n=8 per group) (OGTT determined by two-way ANOVA, AUC determined by two-tailed t-tests). * Comparisons between the BNT group and the saline group. ^#^ Comparisons between the BNT group and metformin treatment (2 mg/kg). **(E-F)** ITT and ABC indexes in *db/db* mice following treatment of mRNA COVID-19 vaccine (4.5 μg/kg) and metformin (300mg/kg) (n=8 per group) (ITT determined by two-way ANOVA, ABC determined by two-tailed t-tests). * Comparisons between the BNT group and the saline group. ^#^ Comparisons between the BNT group and metformin treatment.

**Table S2 Clinical characteristics in healthy controls and subjects with pre-diabetes and diabetes**

| Characteristics | Healthy controls (HC) | | | | |
| --- | --- | --- | --- | --- | --- |
|  | Week0-Baseline (median, IQR) | Week 2-post boosters (median, IQR) | | Changes after boosters (95% CI) | p-value |
| Spike Protein (Log10 pg/mL) | 2.826 (0.399) | 2.915 (0.408) | | 0.1098 (0.0238, 0.1957) | 0.0134 |
| Spike IgG (KU/mL) | 36507 (45542.877) | 157850.996 (127199.561) | | 120810 (99600, 142019) | <0.0001 |
| Neutralization rate (%) | 94.35 （11.214) | 98.022 (0.861) | | 9.582 (5.170, 13.990) | <0.0001 |
| Haemoglobin A1c (HbA1c, %) | 5.3 (0.4) | 5.35 (0.4) | | 0.0180 (-0.0122, 0.0482) | 0.237 |
| Glucose (mmol/L) | 5 (0.5) | 4.9 (0.65) | | -0.0660 (-0.177, 0.0447) | 0.2366 |
| Insulin (pmol/L) | 132.2 (131.209) | 131.424 (114.468) | | -6.388 (-19.66, 6.880) | 0.338 |
| HOMA-IR | 4.594 (4.606) | 4.780 (5.212) | | -0.085 (-0.751, 0.581) | 0.8006 |
| Triglycerides (mmol/L) | 1.055 (0.525) | 1.035 (0.6) | | -0.0162 (-0.153, 0.121) | 0.8135 |
| Triglyceride glucose (TyG) | 8.372 (0.528) | 8.326 (0.644) | | -0.0639 (-0.167, 0.0391) | 0.2185 |
|  |  |  | |  |  |
| Urea (mmol/L) | 4.4 (1.6) | 4.6 (1.1) | | -0.002 (-0.298, 0.2940) | 0.989 |
| Creatinine (μmol/L) | 73.4 (25.125) | 74.15 (27.85) | | -0.786 (-2.649, 1.077) | 0.4006 |
| Bilirubine-total (μmol/L) | 11.4 (6.575) | 12.25 (6.325) | | 0.758 (-0.506, 2.022) | 0.2339 |
| Bilirubine-direct (μmol/L) | 4.35 (2.4) | 4.7 (1.975) | | 0.192 (-0.149, 0.533) | 0.2638 |
| ALP (U/L) | 72 (26) | 75 (26.5) | | -0.1 (-2.961, 2.761) | 0.9443 |
| GGT (U/L) | 22.5 (16.5) | 23 (17.75) | | -0.800 (-4.098, 2.498) | 0.6281 |
| ALT/SGPT(U/L) | 17 (14) | 16.5 (13.25) | | -0.200 (-2.737, 2.337) | 0.8748 |
| AST/SGOT(U/L) | 22 (9.5) | 23 (8.75) | | 1.120 (-0.747, 2.987) | 0.2339 |
| Cholesterol-total (mmol/L) | 5.2 (1.275) | 5.315 (0.985) | | 0.0534 (-0.0583, 0.1651) | 0.3415 |
| HDL-Cholesterol (mmol/L) | 1.32 (0.645) | 1.39 (0.517) | | 0.0356 (-0.0069, 0.0782) | 0.0994 |
| LDL-Cholesterol (mmol/L) | 3.055 (0.9775) | 3.19 (0.985) | | 0.0392 (-0.0656 , 0.1440) | 0.456 |
| C-Peptide (pg/mL) | 931.31 (680.49) | 1005.735 (902.52) | | -5.905 (-134.4, 122.6) | 0.9268 |
| Characteristics | Pre-diabetes (PreD) | | | | |
|  | Week0-Baseline (median, IQR) | | Week 2-post boosters (median, IQR) | Changes after boosters (95% CI) | p-value |
| Spike Protein (Log10 pg/mL) | 2.826(0.450) | | 2.995 (0.441) | 0.1118 (0.0675, 0.1560) | <0.0001 |
| Spike IgG (KU/mL) | 51932.748 (59630.821) | | 176032.565 (142274.453) | 121685 (97945, 145424) | <0.0001 |
| Neutralization rate (%) | 97.207 (6.727) | | 98.411 (0.837) | 4.36 (2.445, 6.274) | <0.0001 |
| Haemoglobin A1c (HbA1c, %) | 6.1 (0.4) | | 6 (0.4) | 0.019 (-0.014, 0.052) | 0.26 |
| Glucose (mmol/L) | 5.65 (0.9) | | 5.5 (0.950) | -0.07 (-0.202, 0.061) | 0.29 |
| Insulin (pmol/L) | 156.318 (129.219) | | 160.794 (118.464) | 4.624 (101.8, 520.5) | 0.334 |
| HOMA-IR | 6.758(6.096) | | 7.130 (6.002) | 1.997 (-0.821, 0.517) | 0.63 |
| Triglycerides (mmol/L) | 1.045 (0.728) | | 1.06 (0.753) | 0.016 (-0.072, 0.103) | 0.72 |
| Triglyceride glucose (TyG) | 8.387 (0.790) | | 8.534 (0.694) | 0.027 (-0.049, 0.102) | 0.485 |
|  |  | |  |  |  |
| Urea (mmol/L) | 5 (2) | | 4.8 (1.925) | -0.0984 (-0.353, 0.156) | 0.443 |
| Creatinine (μmol/L) | 75.15 (26.375) | | 73.85 (24.1) | -0.478 (-2.039, 1.083) | 0.543 |
| Bilirubine-total (μmol/L) | 9.45 (4.375) | | 10.2 (6.125) | 1.823 (-1.105, 4.752) | 0.218 |
| Bilirubine-direct (μmol/L) | 3.9 (1.4) | | 3.8 (1.85) | 0.131 (-0.132, 0.394) | 0.322 |
| ALP (U/L) | 73 (25.5) | | 74 (29.75) | 1.5 (-0.305, 3.305) | 0.102 |
| GGT (U/L) | 30 (22.25) | | 29 (26) | 0.719 (-0.834, 2.272) | 0.359 |
| ALT/SGPT(U/L) | 22 (18.75) | | 20.5 (15.25) | -1.828 (-3.323, -0.333) | 0.017 |
| AST/SGOT(U/L) | 23 (9.25) | | 22 (8.25) | -1.219 (-2.479, 0.041) | 0.058 |
| Cholesterol-total (mmol/L) | 4.655 (1.583) | | 4.7 (1.745) | -0.085 (-0.198, 0.028) | 0.137 |
| HDL-Cholesterol (mmol/L) | 1.38 (0.435) | | 1.37 (0.475) | 0.423 (-0.187, 0.439) | 0.423 |
| LDL-Cholesterol (mmol/L) | 2.68 (1.168) | | 2.78 (1.62) | -0.0475 (-0.147, 0.052) | 0.342 |
| C-Peptide (pg/mL) | 1162.410 (638.548) | | 1156.930 (735.373) | 34.8 (-25.79, 95.39) | 0.2554 |
| Characteristics | Type 2 diabetes (T2D) | | | | |
|  | Week0-Baseline (median, IQR) | | Week 2-post boosters (median, IQR) | Changes after boosters (95% CI) | p-value |
| Spike Protein (Log10 pg/mL) | 2.895 (0.320) | | 2.996 (0.335) | 0.1156 (0.0661, 0.1650) | <0.0001 |
| Spike IgG (KU/mL) | 47133.477 (50230.805) | | 159987.915 (112926.220) | 121685 (97945, 145424) | <0.0001 |
| Neutralization rate (%) | 97.696 (3.420) | | 98.312 (0.957) | 4.206 (1.165, 7.247) | 0.0075 |
| Haemoglobin A1c (HbA1c, %) | 7 (0.775) | | 7.1 (0.7) | 0.0833 (0.0141, 0.153) | 0.019 |
| Glucose (mmol/L) | 7.25 (2.075) | | 7.3 (1.675) | -0.3379 (-0.898, 0.222) | 0.2325 |
| Insulin (pmol/L) | 194.434 (162.010) | | 216.962 (157.247) | 18.56 (4.363, 32.76) | 0.0112 |
| HOMA-IR | 11.108 (9.099) | | 11.741 (9.734) | 1.787 (0.159, 3.415) | 0.0362 |
| Triglycerides (mmol/L) | 1.11 (0.78) | | 1.3 (1.160) | 0.276 (0.051, 0.500) | 0.017 |
| Triglyceride glucose (TyG) | 8.842 (0.713) | | 8.953 (0.764) | 0.166 (0.056, 0.276) | 0.0038 |
|  |  | |  |  |  |
| Urea (mmol/L) | 5.25 (1.07) | | 5.2 (1.475) | -0.0151 (-0.3104, 0.2801) | 0.919 |
| Creatinine (μmol/L) | 70.5 (23.975) | | 73.35 (28.6) | 1.558 (-0.379, 3.394) | 0.113 |
| Bilirubine-total (μmol/L) | 10.55 (7.475) | | 11.65 (6.475) | 3.385 (-1.565, 8.335) | 0.1768 |
| Bilirubine-direct (μmol/L) | 4.3 (2.5) | | 4.6 (2.6) | 0.2545 (-0.092, 0.6009) | 0.147 |
| ALP (U/L) | 83 (37) | | 83 (33) | 2.773 (0.005, 5.540) | 0.0496 |
| GGT (U/L) | 29 (21.75) | | 28.5 (29) | 1.712 (-0.577, 4.002) | 0.1401 |
| ALT/SGPT(U/L) | 22 (22) | | 23.5 (24) | 2.303 (-0.0584, 4.664) | 0.0558 |
| AST/SGOT(U/L) | 21 (9.75) | | 22 (9) | 2.485 (0.328, 4.642) | 0.0246 |
| Cholesterol-total (mmol/L) | 4.14 (0.807) | | 4.315 (0.91) | 0.396 (-0.2604, 1.052) | 0.2327 |
| HDL-Cholesterol (mmol/L) | 1.22 (0.35) | | 1.25 (0.32) | -0.0021 (-0.0299, 0.0257) | 0.8796 |
| LDL-Cholesterol (mmol/L) | 2.37 (0.675) | | 2.315 (0.793) | -0.0546 (-0.2026, 0.09332) | 0.4635 |
| C-Peptide (pg/mL) | 1270.120 (587.575) | | 1393.515 (655.865) | 71.95 (-7.450, 151.3) | 0.075 |

**Table S3 Reagent or Resource used in this study, Related to Materials and Methods**

| Reagent or Resource | SOURCE | IDENTIFIER | STATE AND COUNTRY |
| --- | --- | --- | --- |
| Antibodies |  |  |  |
| Rabbit anti-phospho-Akt (Ser473) | Cell Signaling | Cat# 4058 | Massachusetts, USA |
| Rabbit anti-Akt (1:1000) | Cell Signaling | Cat# 9272 | Massachusetts, USA |
| Rabbit anti-Insulin Receptor β (4B8) | Cell Signaling | Cat# 3025 | Massachusetts, USA |
| Phospho-IGF-I Receptor β (Tyr1131)/Insulin Receptor β (Tyr1146) Antibody | Cell Signaling | Cat# 3021 | Massachusetts, USA |
| Phospho-IKKα/β (Ser176/180) (16A6) Rabbit mAb | Cell Signaling | Cat# 2697 | Massachusetts, USA |
| anti-β-Actin | Cell Signaling | Cat# 4970 | Massachusetts, USA |
|  |  |  |  |
| Chemicals |  |  |  |
| COVID-19 mRNA Vaccine (BNT162B2) | COMIRNATY 复必泰 |  | Mainz, Germany |
| Sinovac COVID-19 Vaccine | Sinovac Biotech |  | Beijing, China |
| 0.9% Sodium Chloride | BIBRAUN. B. Braun Medical |  | Melsungen, Germany |
| Insulin, Human Recombinant | SIGMA | SKU# 91077C | Missouri, USA |
| Insulin Mouse ELISA Kit | Abcam | Cat# ab277390 | Cambridge, UK |
| Glucose | SIGMA | SKU# D9434 | Missouri, USA |
| RIPA buffer | Thermo Fisher | Cat #89900 | Massachusetts, USA |
| BCA Protein Assay Kit | Thermo Fisher | Cat #23225 | Massachusetts, USA |
| EASYpack Protease Inhibitor Cocktail | Roche | SKU# 5892970001 | Basel, Switzerland |
| PhosSTOP | Roche | SKU# 4906845001 | Basel, Switzerland |
| Triglyceride Liquid Reagent for Diagnostic Set | Stanbio | Cat #SB-2100-430 | Texas, USA |
| SARS-CoV-2 (2019-nCoV) Spike Detection ELISA Kit | Sino Biological | Cat #KIT40591 | Beijing, China |
| Mouse Anti-2019 nCoV(S)IgG ELISA Kit | EAGLE | Cat #KBVH015-52 | New Hampshire, USA |
| SARS-CoV-2 Surrogate Virus Neutralization Test (sVNT) Kit | GenScript | Cat #L00847-A | New Jersey, USA |
| Resorcinolnaphthalein | MCE | HY-122445 | New Jersey, USA |
| IAXO-102 | MCE | HY-125171 | New Jersey, USA |
| Propyl pyrazole triol (PPT) | MCE | HY-100689 | New Jersey, USA |
| Spike Trimer (S1+S2) (B.1.1.529 BA.1, Omicron 1 5,372.00 5,372.00 Variant), His-Tag (SARS-CoV-2) Recombinant | BPS Bioscience | Cat# BPS101343 | California, USA |
| Metformin | MCE | HY-B0627 | New Jersey, USA |
| Human C-Peptide ELISA Kit | Abcam | ab260064 | Cambridge, UK |
| Mice Comprehensive Metabolic Panel (Adiponectin; C-peptide) | ImmunoDiagnostics Limited |  | Hong Kong, China |
| Mice Comprehensive Metabolic Panel (TG; TC; HDL-C; LDL-C; FFA) | ImmunoDiagnostics Limited |  | Hong Kong, China |
|  |  |  |  |
| Experimental models |  |  |  |
| Balb/c male mice | NA | NA |  |
| Human subjects | NA | NA |  |
| *db/db* male mice | NA | NA |  |
|  |  |  |  |
| Cell lines |  |  |  |
| 3T3-L1 | ATCC | CL-173 |  |
| C2C12 | ATCC | CRL-1772 |  |
| HepG2 | ATCC | HB-8065 |  |
| Software and algorithms |  |  |  |
| Prism 8 | GraphPad | NA |  |
| ImageJ | NIH | NA |  |
| Others |  |  |  |
| TissueLyser LT | QIAGEN | NA | Hilden, Germany |
| Blood Glucose meters | Accu-Chek | NA |  |
|  |  |  |  |
| Primers |  |  |  |
| *Ers1*-Reverse (5′-3′) mouse | CTTGTGTGTGGACACTCCGT | NA |  |
| *Ers1*-Forward (5′-3′) mouse | AAGAAAGGCACAAGGCACGA | NA |  |
| *Tlr4*-Reverse (5′-3′) mouse | ATGCATGGATCAGAAACTCAGCA | NA |  |
| *Tlr4*-Forward (5′-3′) mouse | AAACTTCCTGGGGAAAAACTCTGG | NA |  |
| *Esr2*-Reverse (5′-3′) mouse | CGCTCAGGGACCGAGGAAAGTACGT | NA |  |
| *Esr2*-Forward (5′-3′) mouse | GTCATGGCTGAGTATTCGTGACGG | NA |  |
| *Ace2*-Reverse (5′-3′) mouse | TGATGAATCAGGGCTGGGATG | NA |  |
| *Ace2*-Forward (5′-3′) mouse | ATTCTGAAGTCTCCGTGTCCC | NA |  |
| *Actb*-Reverse (5′-3′) mouse | TGCTGTCCCTGTATGCCTCTG | NA |  |
| *Actb*-Forward (5′-3′) mouse | TGATGTCACGCACGATTTCC | NA |  |
| *Tmprss2*-Reverse (5′-3′) mouse | AAGTCCTCAGGAGCACTGTGCA | NA |  |
| *Tmprss2*-Reverse (5′-3′) mouse | CAGAACCTCCAAAGCAAGACAGC | NA |  |
